# Inhibitors of histone deacetylase 6 based on a novel 3-hydroxy-isoxazole zinc binding group

**DOI:** 10.1080/14756366.2021.1981306

**Published:** 2021-09-28

**Authors:** Pasquale Linciano, Luca Pinzi, Silvia Belluti, Ugo Chianese, Rosaria Benedetti, Davide Moi, Lucia Altucci, Silvia Franchini, Carol Imbriano, Claudia Sorbi, Giulio Rastelli

**Affiliations:** a Department of Life Sciences, University of Modena and Reggio Emilia, Modena, Italy; b Department of Precision Medicine, University of Campania “Luigi Vanvitelli”, Naples, Italy; c Biogem Institute of Molecular and Genetic Biology, Ariano Irpino, Italy

**Keywords:** HDAC6 inhibition, zinc-binding-group, HDAC inhibitors, drug design

## Abstract

Histone deacetylase 6 (HDAC6) is an established drug target for cancer treatment. Inhibitors of HDAC6 based on a hydroxamic acid zinc binding group (ZBG) are often associated with undesirable side effects. Herein, we describe the identification of HDAC6 inhibitors based on a completely new 3-hydroxy-isoxazole ZBG. A series of derivatives decorated with different aromatic or heteroaromatic linkers, and various cap groups were synthesised and biologically tested. *In vitro* tests demonstrated that some compounds are able to inhibit HDAC6 with good potency, the best candidate reaching an IC_50_ of 700 nM. Such good potency obtained with a completely new ZBG make these compounds particularly attractive. The effect of the most active inhibitors on the acetylation levels of histone H3 and α- tubulin and their anti-proliferative activity of DU145 cells were also investigated. Docking studies were performed to evaluate the binding mode of these new derivatives and discuss structure-activity relationships.

## 1. Introduction

Histone deacetylases (HDACs) are post-translational enzymes that remove acetyl groups from lysine residues, thereby regulating key processes, such as gene expression^1^. Eleven HDAC isoforms, clustered into four classes, are currently described. HDAC6 is a histone deacetylase isoform belonging to class IIb that contains two catalytic domains and, unlike other HDACs with nuclear localisation, is found mainly in the cytoplasm^2^. Importantly, HDAC6 deacetylates non-histone proteins, such as tubulin, Hsp90 and cortactin^3^.

Several HDAC inhibitors (HDACi) have been developed so far, mainly active against cancer and neurodegenerative diseases, resulting in five approved drugs^4^. The vast majority of the developed inhibitors contain a hydroxamic acid zinc binding group (ZBG). However, several studies have shown that hydroxamic acids are genotoxic due to the Lossen rearrangement of the O-ester, which yields a reactive isocyanate able to covalently modify various cellular components^5^. This undesirable effect motivates the search for HDAC inhibitors with alternative ZBGs.

**Scheme 1.**
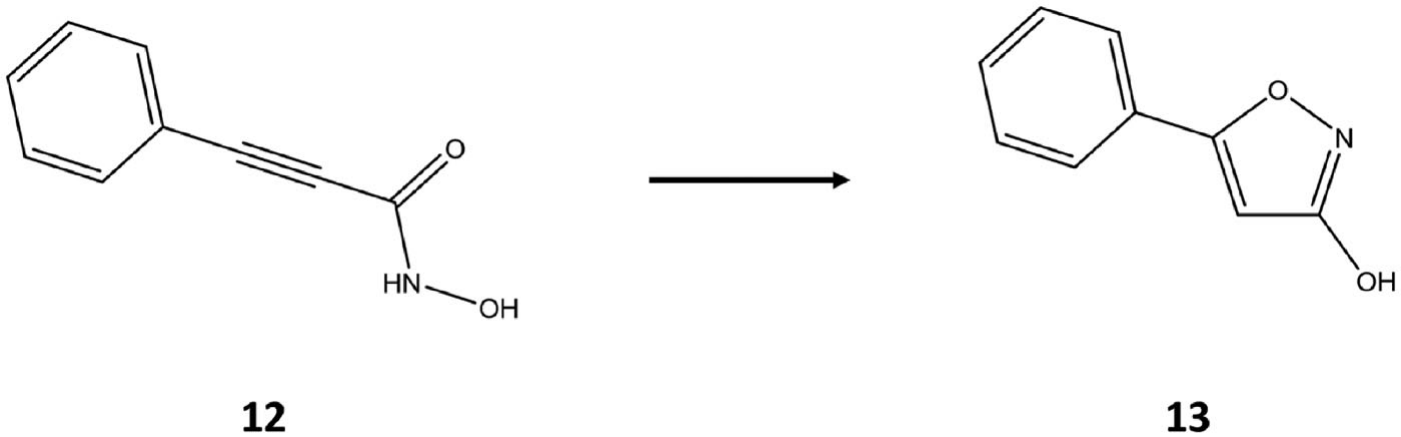
Conversion of the N-hydroxy-3-phenyl-propiolamide **12** into the 3-hydroxy-5-phenyl-isoxazole **13**.

In a recent study, we described the effect of different linker chemotypes on the potency and selectivity of a series of HDACi carrying a hydroxamate ZBG^6^. Interestingly, during the synthesis of N-hydroxy-3-phenyl-propiolamide **12**, we observed the formation of 5-aryl-3-hydroxy-isoxazole **13**, presumably formed through intramolecular cyclisation by 1,4 addition of the hydroxyl group to the conjugated alkyne bond (Scheme 1). Compound **12** converted into **13** over time, even when stored at solid state both at room temperature and at −20 °C, demonstrating that compound **13** is stable. Being constituted by a small heteroaromatic ring with hydrogen bond acceptor and donor atoms, we hypothesised that the 3-hydroxy-isoxazole moiety could satisfy the pharmacophore requirements necessary for the coordination of the catalytic zinc ion of HDAC6. Therefore, in this work we investigated a series of 3-hydroxy-isoxazole derivatives bearing different aromatic or heteroaromatic linkers and various cap groups.

## 2. Results

### 2.1. Ligand-based analyses

We firstly investigated the tautomeric preference of compound **13** in water solvent by means of quantum-chemical calculations, performed with Jaguar of the Schrödinger suite 2020–1, with default settings^7^^,^^8^. Indeed, five-membered heteroaromatic rings, such as 3-hydroxy-isoxazole, could undergo keto–enol tautomerism in water^9^^,^^10^. Results of the quantum-chemical calculations indicated that the enol form is 0.5 kcal/mol more stable than the keto tautomer, which is likely to be favoured by aromaticity. This result is in line with previous *in silico* and experimental studies on the tautomeric equilibria of 3-hydroxy-substituted isoxazoles^10^, suggesting that the 3-hydroxy tautomer is preferentially present in solution. Therefore, all *in silico* calculations were conducted with the enol form of the ligands.

**Figure 1.**
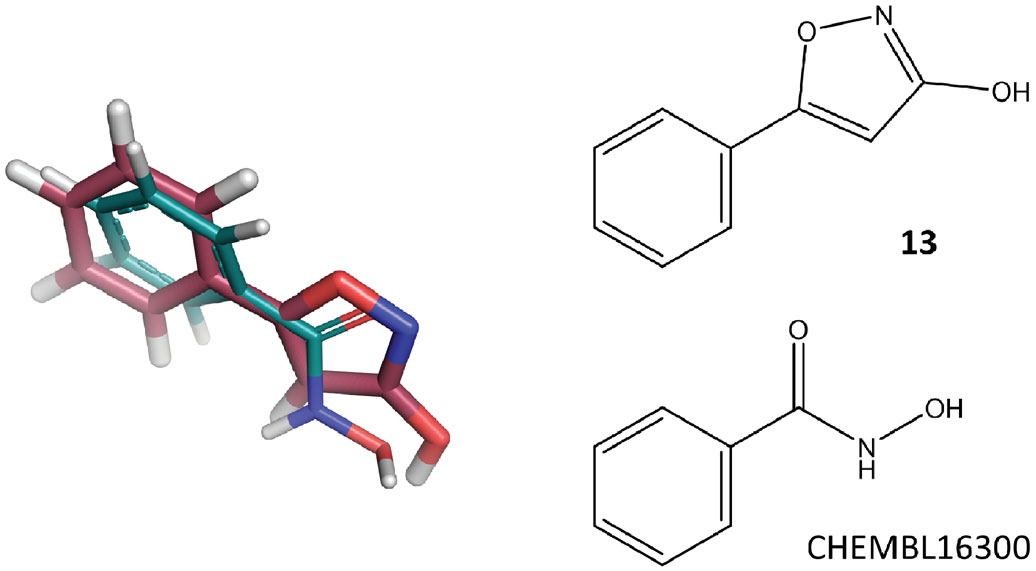
3D ligand-based alignment of **13** with respect to the known HDAC6 inhibitor ChEMBL16300. **13** and ChEMBL16300 are represented as raspberry and deep teal sticks, respectively.

To evaluate whether compound **13** could provide a valuable bioisosteric replacement of previously reported HDAC6 ZBGs^11^, extensive ligand-based similarity analyses were performed in the ChEMBL database^12^. To this aim, HDAC6 ligands reported in ChEMBL (accessed on 29 May 2020) were subjected to a multi-conformer *vs* multi-conformer 3D ligand-based virtual screening, by using the ROCS software^13^. Moreover, a series of 2D ligand-based analyses was carried out by using the MACCS and ECFP4 molecular fingerprints implemented in the OpenEye *Python Toolkits* (version 2020.1.0)^14^. Experimental details on the performed ligand-based analyses are reported in the Supplementary material. These analyses highlighted a significant degree of similarity between compound **13** and several ChEMBL ligands bearing the hydroxamate ZBG (see Table S1 of the Supplementary material). For example, visual inspection of the predicted ligand alignments showed that compound **13** overlaps very well with the benzhydroxamic acid inhibitor ChEMBL16300 (IC_50_ = 115 nM), resulting in a similar location of the two phenyl rings and a good alignment of hydrogen bond donor/acceptor groups of the hydroxamic acid and the 3-hydroxy-isoxazole (Figure 1). Moreover, some of the identified ChEMBL ligands have been reported to bind to the Zn^2+^ ion with a bidentate coordination in crystallographic complexes of HDAC6 (Table S1). Altogether, these results suggest that the 3-hydroxy-isoxazole could represent a valuable bioisosteric replacement of the hydroxamic acid moiety.

### 2.2. Chemical synthesis

**Scheme 2.**
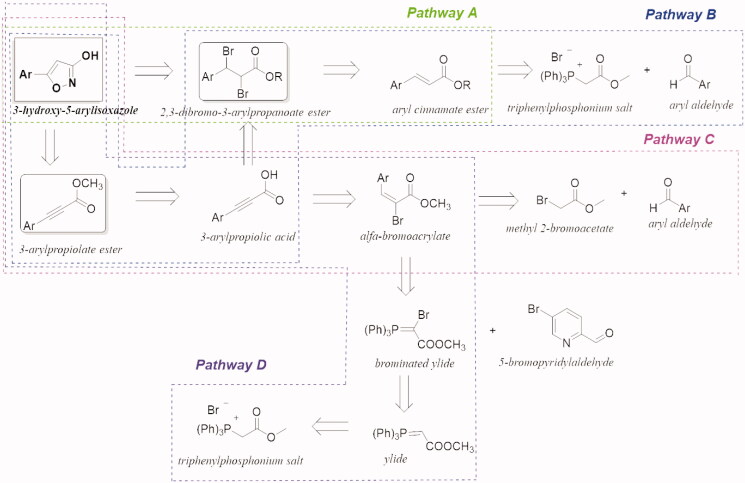
Retrosynthetic strategies adopted for the synthesis of the 3-hydroxy-5-arylisoxazoles.

**Figure 2.**
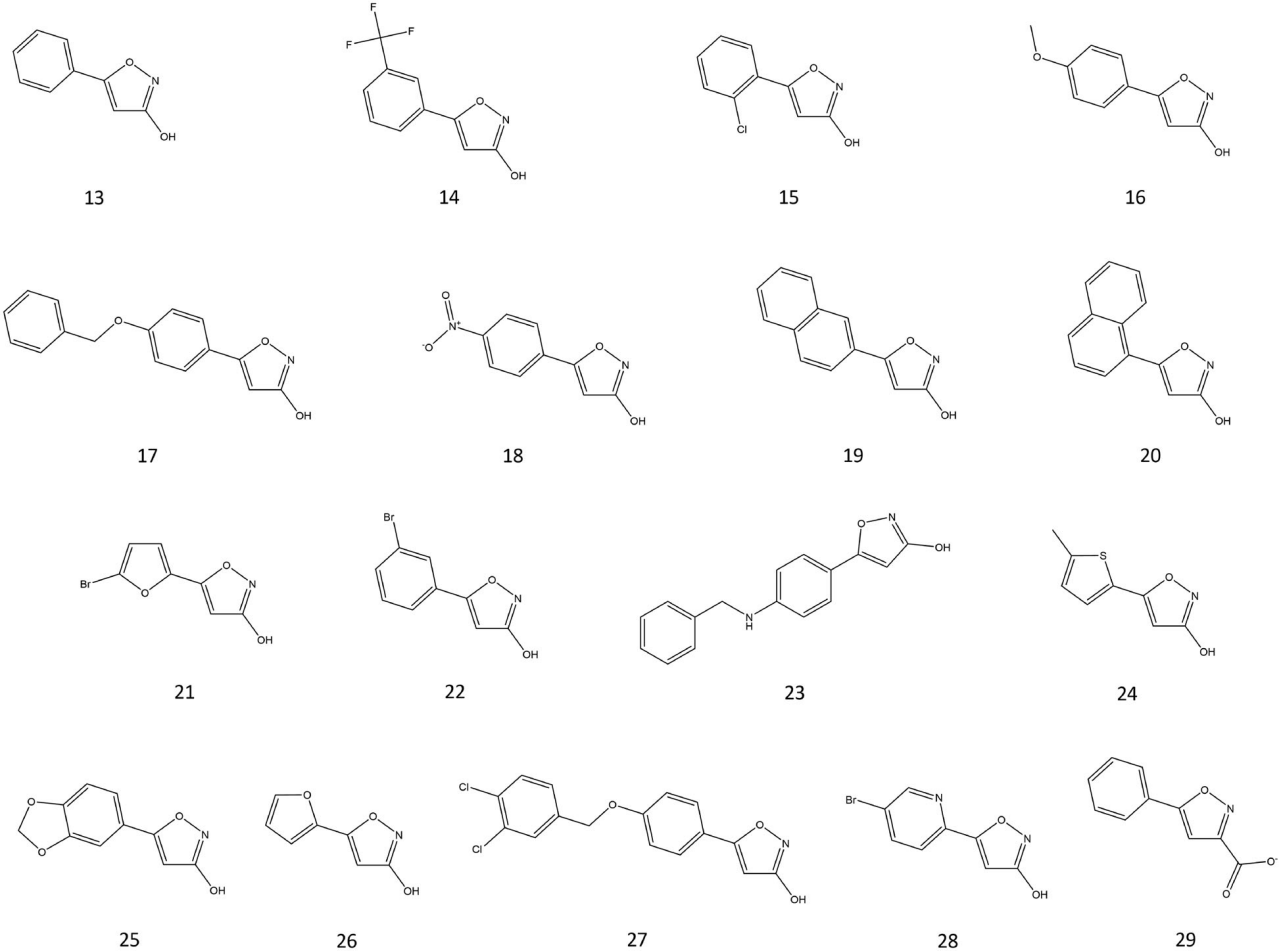
Chemical structures of the investigated 3-hydroxy-5-arylisoxazole compounds.

 We therefore synthesised and *in vitro* tested a small series of compounds based on the 3-hydroxy-isoxazole scaffold. Modifications included different linker portions (aromatic and heteroaromatic) and the introduction of selected cap groups. The synthesised compounds are reported in Figure 2. With the exception of **13** and **29**, all the other 3-hydroxy-5-aryl/heteroaryl-isoxazole derivatives were synthesised according to the retrosynthetic strategies depicted in Scheme 2. All the experimental synthetic procedures and the discussion of each synthetic pathway are reported in the Supplementary material, together with the synthetic Schemes S1–S6.

**Scheme 3.**
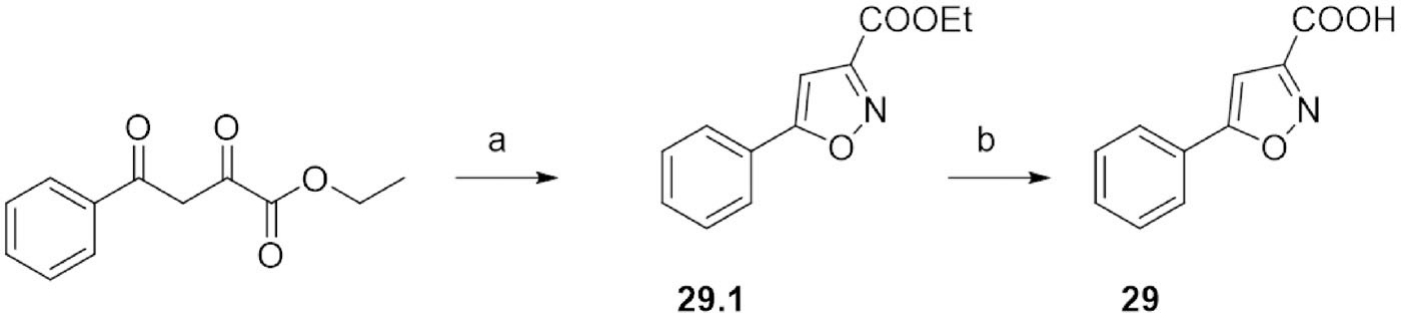
Synthesis of **29**.

As a first attempt, the 3-hydroxy-5-arylisoxazole scaffold was directly obtained by cyclisation from hydroxylamine and the 2,3-dibromo-3-arylpropanoate ester following the procedure of Panda et al.^15^, as reported in the Pathway A. The 2,3-dibromo-3-arylpropanoate ester was readily prepared by brominating the appropriate aryl cinnamate ester, which was previously synthesised by esterification of the corresponding aryl cinnamic acid. This synthetic pathway provided easily the ester intermediate with high yield. On the contrary, the last step gave low yields, probably due to the harsh reaction conditions, which produced several and unidentified by-products, making the subsequent purification procedure particularly hardworking. Pathway B resulted in cyclisation of the appropriate 3-arylpropiolate ester with hydroxylamine using milder reaction conditions^16^. The 3-arylpropiolate ester derives from the corresponding acid, which is obtained from the 2,3-dibromo-3-arylpropanoate ester by a dehydrohalogenation reaction. The dibromo-derivative was in turn obtained from bromination of an aryl cinnamate ester, which was produced by Wittig reaction between the appropriate aryl aldehyde and the triphenylphosphonium salt. By using this synthetic pathway, the final desired products were obtained with high yield and purity. However, the main limitations of this procedure are the low commercial availability of various 3-arylpropiolate esters and their limited synthetic accessibility. In this regard, besides the Sonogashira’s reaction followed in the past, a second strategy for the synthesis of the 3-arylpropiolate esters was adopted. It requires a dehydrohalogenation of the corresponding 2,3-dibromo-derivative, using alcoholic KOH, then a re-esterification of the obtained 3-arylpropanoic acid. However, although this retrosynthetic pathway adds two synthetic steps and the strong alkaline conditions of dehydrohalogenation lead to the formation of by-products, the overall yield was higher than that obtained following the previously described Pathway A. Moreover, during the bromination reaction of the methyl 3–(5-methyltiophen-2-yl)acrylate, in addition to the desired dibromo-derivative, an α-bromoarylacrilate (methyl 2-bromo-3–(5-methylthiophen-2-yl)acrylate) was isolated and identified. This compound gave us the opportunity to follow another approach for the synthesis of the 3-arylpropiolate esters (Pathway C). According to Kim et al.^17^ α-bromoacrylate can be converted into the corresponding 3-arylpropiolate ester using a mixture of sodium amide (NaNH_2_) and potassium *tert*-butoxide (*t*-ButOK) in a non-protic solvent (i.e. THF). This procedure, tested on the methyl 2-bromo-3–(5-methylthiophen-2-yl)acrylate, allowed us to obtain the 3–(5-methylthiophen-2-yl)propiolic acid with quantitative yield and without by-products. Thus, the next 3-arylpropiolate esters were prepared accordingly to the improvements achieved following Pathway C. The appropriate 2-bromo-3-arylacrylate esters were easily synthesised by reacting the arylaldehydes with 2-bromoacetate esters in the presence of titanium tetrachloride (TiCl_4_) and triethylamine (TEA), as reported by Augustine et al.^18^. This procedure was successfully applied for the synthesis of diverse aryl and heteroaryl α-bromo-acrylates and gave high yields. Unfortunately, starting from pyridyl carboxaldehyde this synthetic procedure was not effective. Therefore, for the synthesis of the methyl 2-bromo-3–(5-bromopyridin-2-yl)acrylate a variant of the Wittig’s reaction was adopted (Pathway D)^19^. The 5-bromopyridin-2-yl carboxaldehyde was condensed with the brominated ylide to give directly the α-bromopyridyl acrylate. The brominated ylide was prepared by reacting the triphenylphosphonium salt with NaOH to obtain the ylide, which was successively mono-brominated with bromine. A last and different synthetic approach was used to obtain the 5-phenylisoxazole-3-carboxylic acid **29**. The ethyl 2,4-dioxo-4-phenylbutanoate was cyclized with hydroxylamine hydrochloride (a) and the obtained ethyl ester **29.1** was then hydrolysed with NaOH 1 M (b, Scheme 3).

Since the synthesised compounds have not be subjected to *in vivo* assays, their purity was evaluated by NMR analysis (>90%). In this regard and as an example, the 1H NMR spectra images of the most interesting derivatives (i.e. **17**, **23**, **25** and **27**) have been reported, together with that of the first compound (**13**) of this series (see Supplementary material).

### 2.3. Biological evaluation

The obtained compounds were tested *in vitro* for the inhibition of recombinant human HDAC6, through dose-response assays performed in the 300 µM to 135 nM concentration range. The results are reported in Table 1, and dose-response curves are reported in Figure S1. While **14–16**, **19**, **20** and **29** were inactive, displaying more than 55% HDAC6 activity at the highest tested concentration (300 µM), compounds **13, 22**, **26** and **28** were scarcely active showing IC_50_ values of 98.1, 73.3, >100, and 82.3 µM, respectively. We identified three active molecules with IC_50_ values ranging from 10 to 50 µM (**18**, **21**, and **24**), three compounds (**17**, **25**, **27**) with IC_50_ values below 10 µM and one compound (**23**) with IC_50_ below 1 µM. The most active compound (**23**) reached an IC_50_ of 700 nM. The most active compounds were further tested on the acetylated p53 peptide -residues 379–382 (RHKK^Ac^AMC)- as substrate for HDAC6. The results corroborate the inhibitory activity of **17**, **23**, **25** and **27** at concentrations lower than 10 µM. The most active compounds **17**, **23**, **25** and **27** were tested *in vitro* in DU145, an androgen insensitive prostate cancer cell line (PCa) with very low or undetectable AR levels, which represents the castration-resistant PCa (CRPC) model. HDACs contribution to CRPC is known^20^ and the possibility to arrest CRPC proliferation by acting on HDAC targets is expanding in the scientific literature^21^^,^^22^. After administration of the selected compounds for 24 h at 10 µM concentration, the effect on the acetylation levels of histone H3 and α-tubulin (respectively, HDAC1 and HDAC6 targets) was investigated. While no effect was observed on the acetylation levels of histone H3 (Figure S3), the acetylation of α-tubulin increased after treatment with **23** and **25**, suggesting a potential HDAC6 selectivity for both compounds. Lastly, in the same cell line, the anti-proliferative effect was measured in dose and time dependent manner (Figure S4). As shown in Figure S4, the compounds reduced the proliferation rate of DU145 cells at a 50 µM concentration. In particular, **23**, **25** and **27** exerted anti-proliferative activity in late phases at the lowest concentrations as well.

**Table 1.** Inhibitory activity (IC_50_) of the investigated 3-hydroxy-isoxazole compounds on HDAC6.

Compound ID	HDAC6 IC_50_ (µM)
Trichostatin A	0.026
13	98.1
14	*n.a*.
15	*n.a*.
16	*n.a.*
17	1.3
18	16.4
19	*n.a*.
20	*n.a*.
21	29.6
22	73.3
23	0.7
24	21.9
25	1.5
26	>100
27	8.2
28	82.3
29	*n.a*.

*n.a.*: not active. The compound displayed more than 55% enzyme activity at the highest tested concentration (300 µM).

### 2.4. Molecular docking

**Figure 3.**
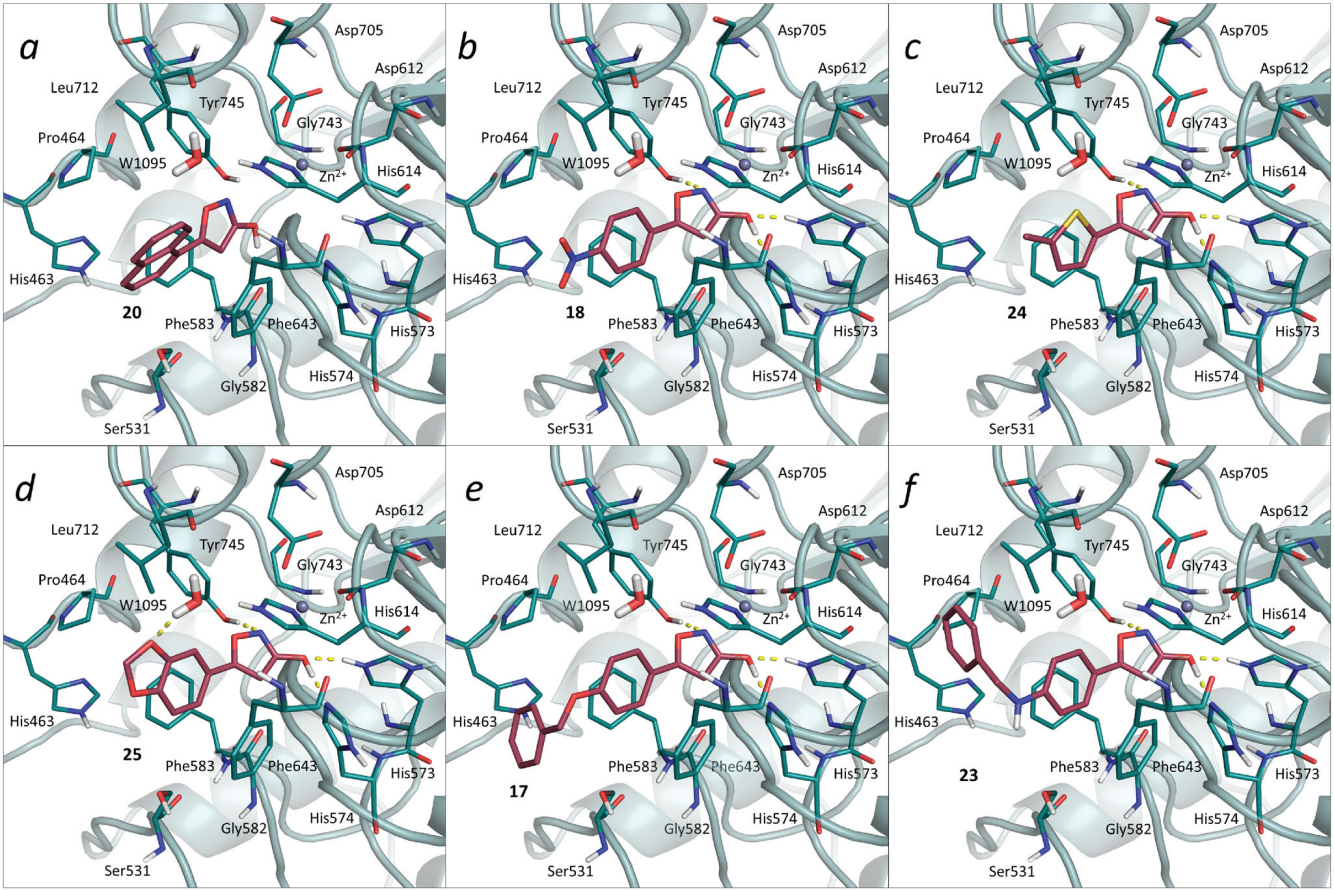
Predicted docking poses for representative the 3-hydroxy-isoxazole compounds into the HDAC6 binding site. Panels *a*–*h* report the predicted binding modes of compounds **20**, **18**, **24**, **25**, **17**, and **23** in 5WGI (HDAC6), respectively.

To rationalise the structure-activity relationships (SAR) of the reported compounds, docking calculations were performed into a representative HDAC6 crystal structure (PDB code: 5WGI^23^). The experimental details are reported in the Supplementary material. Docking calculations were performed with the Glide software of the Schrödinger suite 2020–1^24^. According to the predicted binding modes, the 3-hydroxy-isoxazole moiety establishes a bidentate coordination with the catalytic Zn^2+^ of the HDAC6 active site, similarly to other previously reported ligands with different ZBGs^23^^,^^25–27^. In particular, the compounds under investigation were predicted to coordinate the catalytic zinc ion with the 3-hydroxy group, and the nitrogen and oxygen atoms of the isoxazole ring. Moreover, the 3-hydroxyl group established a hydrogen bond interaction with the His573 in the protonated form, similarly to Trichostatin A (TSN) in the 5WGI crystal complex^23^^,^^28^. Interestingly, docking calculations performed in the 5WGI structure with a neutral His573 provided similar results, the Root Mean Square Deviations (RMSD) values observed for the respective predicted poses being below 2.0 Å (Table S2). This result, which is due to the ability of the 3-hydroxyl group to establish both H-bond acceptor and donor interactions, suggests that the His573 protonation state does not significantly affect the binding mode of the investigated ligands. Likewise, docking results obtained with negatively charged ligands potentially originating from the dissociation of the 3-hydroxyl group were fully comparable, the RMSD obtained between the poses being always below 1.5 Å (Table S2). According to the predicted binding poses, **29**, which presents a carboxylic acid group at position 3 of the isoxazole ring and turned out to be inactive in our *in vitro* assays (Table 1), did not establish interactions with the zinc ion of HDAC6. Moreover, compounds with sterically hindered linkers, such as **19** and **20** (Figure 3, *panel a*), could not be accommodated in the catalytic tunnel of the histone deacetylase, due to steric clashes with the Phe643, Phe583 and His614 side chains. Similar considerations apply also for **15**, which is substituted with an ortho chlorine substituent. Compounds with a 4-substituted phenyl (e.g. **18**) (Figure 3, *panel b*) or 5-methyl-thiophene group (i.e. **24**) (Figure 3, *panel c*) are able to establish favourable π-π stacking interactions with the side chains of the residues lining the catalytic tunnel. Ligands with polar substituents in meta position, such as **14**, could not establish a favourable coordination of the HDAC6 catalytic zinc, thus resulting inactive or significantly less active.

In compound **25**, one of the most active ligands of the series (Table 1 and Figure 3, *panel d*), the 1,3-benzodioxolane moiety establishes favourable π-π stacking interactions with Phe643, Phe583 and His614, and a hydrogen bond interaction with the 1095 water molecule. The latter interaction, observed also in other crystallographic complexes, appears to contribute significantly to the binding of ligands to HDAC6. The furan derivative **26** was almost as active as the phenyl derivative **13**. However, substitution with a bromine, such as in **21** and **22**, provided slightly better activities. Compounds bearing flexible aromatic cap groups, such as **17** (Figure 3, *panel e*) and **27**, were among the most active of the series (Table 1). According to the predicted binding modes, the distal aromatic portion of these molecules establishes favourable hydrophobic interactions with the side chains of Phe583 and Pro464, similarly to the crystallographic ligand in the complex. Indeed, superimposition of the docking poses of **17** and **27** with HDAC6 crystallographic ligands revealed structural details important for the activity. In particular, compounds **17** and **27** present a methyl-ether linker, which is often replaced, in potent HDAC6 inhibitors, by chemical moieties potentially able to interact with Ser531 and/or water molecules in the proximity of the cap region. Notably, the substitution of the methyl-ether linker in **17** with methylamine (i.e. **23** – Figure 3, *panel f*) resulted in a slight improvement of HDAC6 inhibitory activity (Table 1). Altogether, the performed *in silico* analyses helped to rationalise the inhibitory activity of the investigated compounds.

## 3. Conclusions

In conclusion, we have reported a series of HDAC6 inhibitors bearing a completely new 3-hydroxy-isoxazole zinc binding group. Some of the compounds had good potency, the most active one having an IC_50_ of 700 nM, and showed also anti-proliferative activity in prostate cancer cells. In particular, the results of the *in vitro* assays on recombinant proteins allowed us to identify **17**, **23** and **25** as the most interesting candidates of the series. Molecular docking and ligand similarity calculations allowed us to predict their binding mode and discuss the SARs of the reported ligands. Interestingly, the compounds present good activity and low molecular weight. Most importantly, they are based on an entirely new zinc binding group, thus representing an interesting alternative to classical hydroxamic acids. Therefore, they constitute valuable candidates for further preclinical optimisation of HDAC6 inhibitors based on a novel zinc binding group.

## Supplementary Material

Supplemental Material
