# Supplementary material for: Inhibitors of histone deacetylase 6 based on a novel 3-hydroxy-isoxazole zinc binding group
Source: J Enzyme Inhib Med Chem. 2021 Sep 28;36(1):2080–6. doi: 10.1080/14756366.2021.1981306 (PMC8480759; doi:10.1080/14756366.2021.1981306)
Supplement: Supplemental Material [file IENZ_A_1981306_SM0084.pdf]

Supplementary Material

Inhibitors of Histone Deacetylase 6 based on a novel 3-hydroxy-isoxazole zinc binding group.

P. Linciano,[a,Ω,†] L. Pinzi,[a,†], S. Belluti,[a] U. Chianese,[b] R. Benedetti,[b] D. Moi,[a] L. Altucci,[b,c] S. Franchini,[a] C. Imbriano,[a] C. Sorbi,[a,*] and G. Rastelli[a]*

[a] *Department of Life Sciences, University of Modena and Reggio Emilia. Via Campi 103, 41125 Modena, Italy;* [b] *Department of Precision Medicine, University of Campania "Luigi Vanvitelli". Via L. De Crecchio 7, 80138 Naples, Italy;* [c] *Biogem Institute of Molecular and Genetic Biology, Via Camporeale Area P.I.P., 83031 Ariano Irpino, Italy.*

[†] These authors contributed equally.

[Ω] Present address: Department of Drug Sciences, Medicinal Chemistry and Pharmaceutical Technology Section, University of Pavia, V.le Taramelli 12, 27100 Pavia, Italy.

Corresponding authors

* Prof. Giulio Rastelli
Department of Life Sciences
University of Modena and Reggio Emilia.
Via Campi 103, 41125 Modena, Italy.
Phone: +39 0592058564; E-mail: giulio.rastelli@unimore.it

* Dr. Claudia Sorbi
Department of Life Sciences
University of Modena and Reggio Emilia.
Via Campi 103, 41125 Modena, Italy.
Phone: +39 0592058583; E-mail: claudia.sorbi@unimore.it

TABLE OF CONTENT

Tables
Table S1…… S3
Table S2…… S4

Figures
Figure S1…… S5
Figure S2…… S6
Figure S3…… S7
Figure S4…… S8

Synthetic Pathways and Schemes
Pathway A and Scheme S1…… S14
Pathway B, and Schemes S2 and S3…… S15
Pathway C and Scheme S4…… S17
Pathway D and Scheme S5…… S18
Pathway E and Scheme S6…… S19

Experimental Methods
1.1 Molecular Modelling…… S9
1.2 Synthetic Procedures…… S13
1.3 In Vitro HDAC6 Assay…… S36
1.4 Western blot analysis…… S36
1.5 Anti-proliferative assay…… S37

References …… S38

Table S1. HDAC6 ligands of ChEMBL identified as the most similar to compound **13**, according to the performed ligand-based analyses.

Compound ID	*ChEMBL ligand*	*Ligand similarity*			*ECFP4fp*	*MACCSfp*	*HDAC6 IC_{50} (nM)*[a]
		Tanimoto Combo	*Shape Tanimoto*[b]	*Color Tanimoto*			
13	CHEMBL152665	1.176	0.848	0.328	0.311	0.217	1100
13	CHEMBL16300	1.06	0.862	0.198	0.359	0.238	115
13	CHEMBL2333343	0.971	0.813	0.158	0.333	0.190	28
13	CHEMBL2333344	1.062	0.859	0.202	0.277	0.14	12
13	CHEMBL2333345	1.051	0.852	0.2	0.300	0.17	376
13	CHEMBL2333346	0.977	0.834	0.142	0.310	0.18	30
13	CHEMBL2381522	1.049	0.852	0.198	0.350	0.291	650
13	CHEMBL3415449	1.089	0.821	0.268	0.320	0.190	1770

Note: [a] activity data was retrieved from ChEMBL (accessed on May 29th, 2020).
[b] compounds with Shape Tanimoto values higher than 0.75 are considered similar, according to literature data[21].

Table S2. Root-mean-squared deviation (RMSD) between the poses predicted by docking on 5WGI with His573 in its protonated form. Docking calculations were performed in this receptor for both the neutral and negatively charged forms of the investigated compounds.

Compound ID	***RMSD Neutral compounds (Å)***	***RMSD Deprotonated compounds (Å)***
13	0.15	0.17
14	0.11	0.15
15	0.13	0.24
16	0.06	0.16
17	0.93	1.07
18	0.16	0.14
19	0.29	0.10
20	0.05	0.14
21	0.19	0.21
22	0.23	0.11
23	0.1	0.16
24	0.06	0.24
25	0.1	0.16
26	0.1	0.19
27	1.59	0.88
28	0.04	0.16
29[a]	0.19	

Note: compound 29 was docked only in its negatively charged form.

Figure S1. Dose-response curves of the investigated HDAC6 inhibitors.

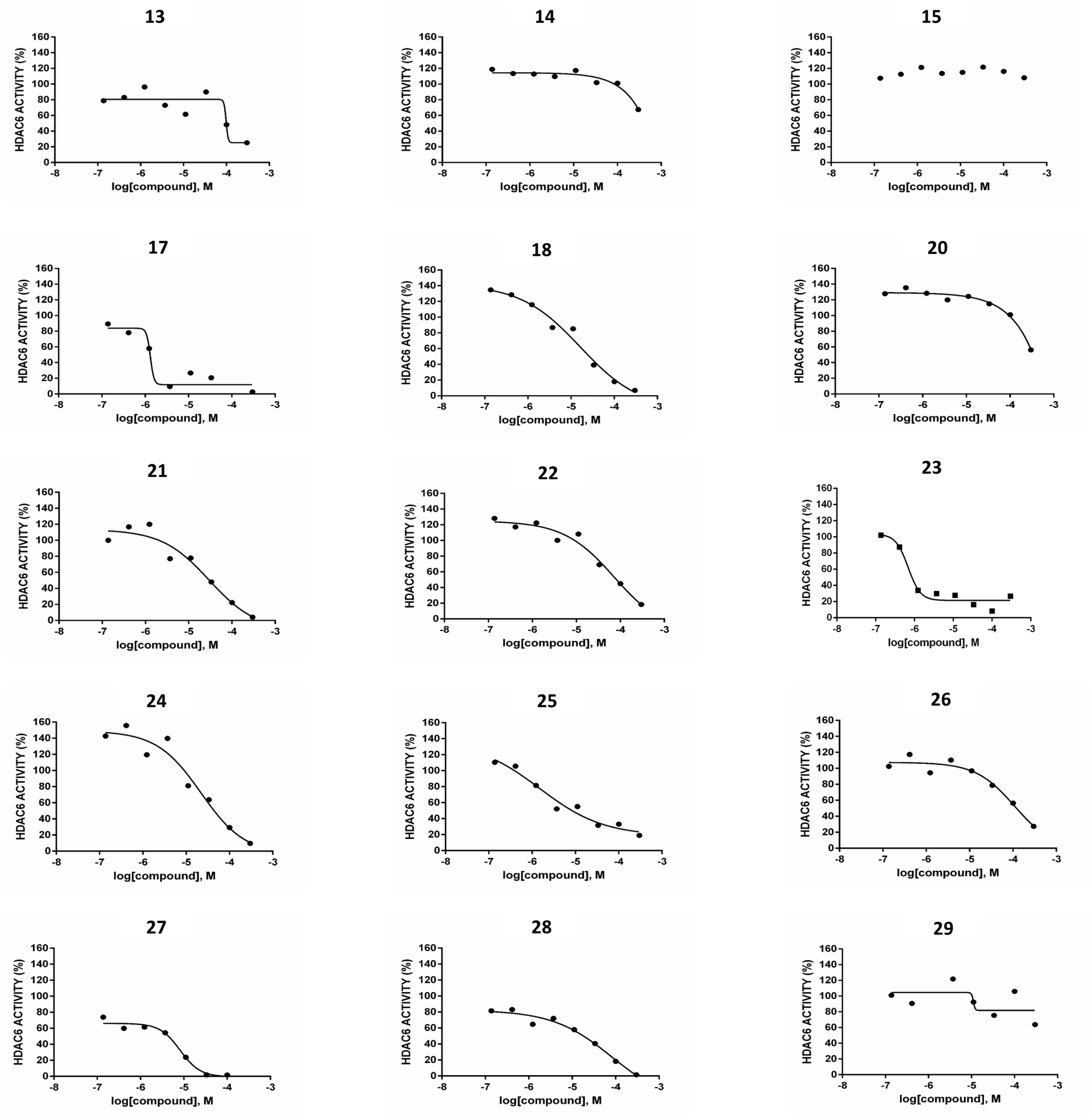

Figure S2. Docking of **26** into the HDAC6 binding site. The pharmacophoric features of the HDAC6 biding side, which were mapped with the SiteMap software (Schrödinger), are also depicted to better clarify the ligand-protein complementarity of **26**. HDAC6 binding site residues and **26** are represented as deep teal and grey sticks, respectively. H-bond acceptor, H-bond donor and hydrophobic features are represented in red, blue and yellow surfaces, respectively. The image was created with Maestro (Schrödinger release 2020-1).

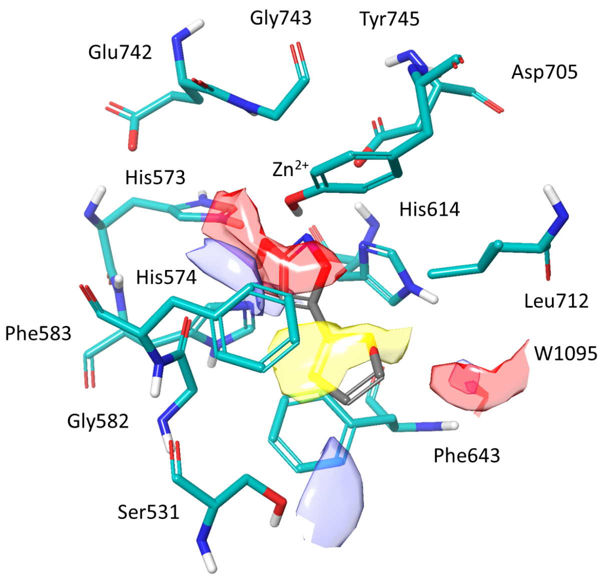

Figure S3. Western blotting analysis of the tested and reference compounds SAHA and Tubastatin A on acetylation levels of histone H3 and α-tubulin in DU145 cells. GAPDH and H4 were used as a loading control. Densitometric values were analyzed using ImageJ Software and are reported on the top of bands as the ratio between loading control and acetylated target.

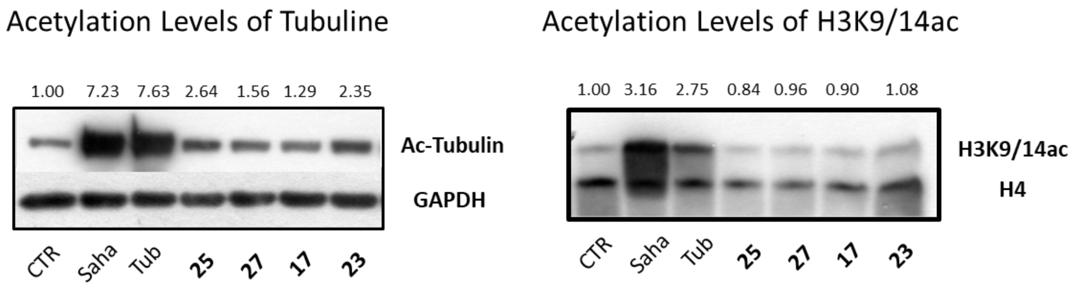

Figure S4. Cell viability assay (MTT) of the tested compounds in human prostate cancer cell lines (DU145). Cells were treated with 4 increasing concentrations (1, 5, 10, and 50 μM) of tested compounds, monitored at 24, 48, and 72 hours and tested with SAHA and Tubastatin A as reference compounds. Data are expressed as mean ± SEM.

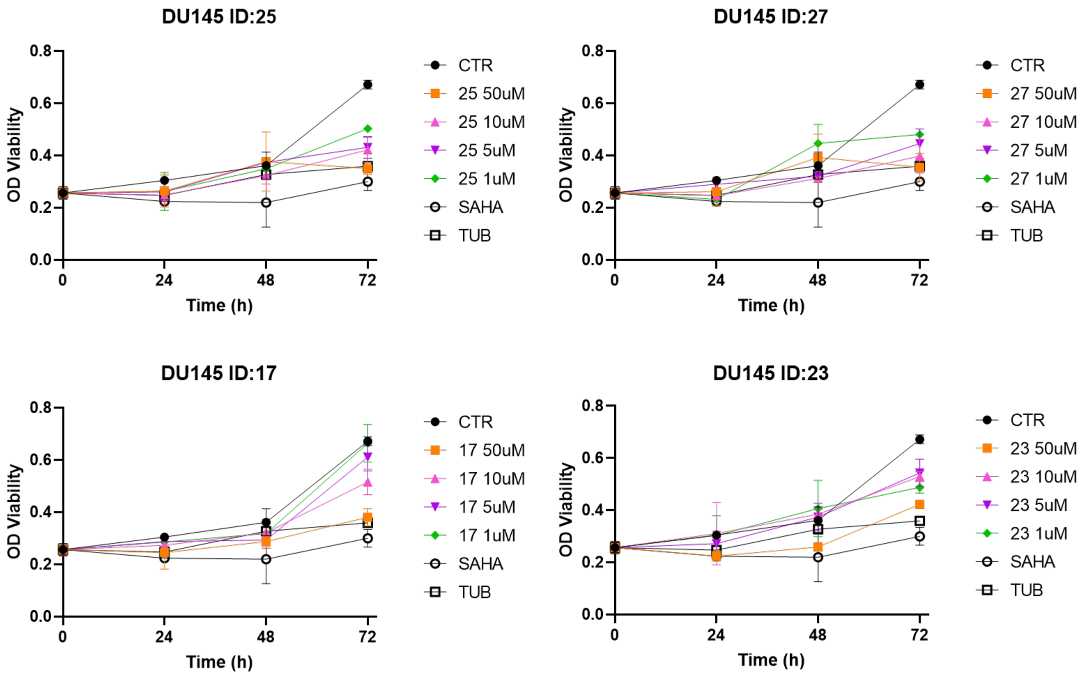

1. Experimental Methods

1.1 Molecular modelling

Ligand-based analyses

To evaluate whether the 3-hydroxy-isoxazole moiety could serve as an efficient bioisosteric replacement of already reported HDAC6 zinc-binding groups, 3D and 2D ligand-based similarity analyses were performed between compound **13** and HDAC6 ligands reported in the ChEMBL database[1]. To this aim, HDAC6 ligands were firstly downloaded from the ChEMBL database (accessed on: May 29th, 2020), and then filtered to retain only those with activity data reported on human proteins, in terms of IC_{50}, K_i, K_d, EC_{50} or % of inhibition. Afterwards, compound **13** and HDAC6 ligands previously filtered were prepared for the ligand-based analyses as follows. The ligands were pretreated by using *LigPrep*[2], with default settings, and then, and then processed with the OMEGA2 software[3,4]. This procedure allowed to obtain up to 5 conformers for the compound bearing the 3-hydroxy-isoxazole scaffold under investigation and a database of multi-conformers HDAC6 ligands, each containing up to 50 different conformations. Afterwards, a multi-conformer *vs* multi-conformer 3D shape-based virtual screening was performed by using the ROCS software[5,6]. Default settings were used to perform the 3D similarity screenings, except for the "*subrocs*" parameter, which allows to more accurately evaluate ligand alignments at a reasonable computational cost, especially when the query and database molecules might have a large difference in size.

2D ligand-based analyses were also performed by using *in house* developed scripts, implemented in the *OpenEye Python Toolkits* (release 2020-1)[7]. In particular, the 2D ligands similarity was evaluated by using the MACCS and ECFP4 fingerprints that allow to describe different structural features of the molecules, the degree of similarity between compounds being evaluated in terms of the Tanimoto score[8].

Visual inspection of the predicted 3D ligand-based alignments was restricted to the first 500 best scoring compounds according to the Tanimoto Combo score (default). Moreover, compounds with similarity scores below 0.3 (MACCS) and 0.8 (ECFP4)[9], were not visually inspected.

Structure-based analyses

X-ray crystal structures of *human* and *danio rerio* histone deacetylase 6 (HDAC6), were firstly downloaded from the Protein Data Bank (accessed on May 29th, 2020)[10], and then aligned with the "*Protein Structural alignment*" utility of the Schrödinger Suite 2020-1[11]. The complete list of the collected PDB crystal structures is reported in Table A.

Table A. HDAC6 CDII crystal structures collected from the PDB.

PDB code	*PDB chain*	*Type of ligand linker*	*Water molecules near the CAP*	*Ligand Zinc Binding Group*	*Water molecules near the Zn^{2+}*	*Resolution (Å)*
5EDU[a]	A	alkyl		hydroxamate		2.79
5EDU[a]	B	alkyl		hydroxamate		2.79
5EEI	A	alkyl	YES	hydroxamate		1.32
5EEI	B	alkyl	YES	hydroxamate		1.32
5EEK	A	alkyl	YES	hydroxamate		1.59
5EEM	A					2.00
5EEM	B					2.00
5EEN	A	alkyl	YES	hydroxamate		1.86

5EEN	B	alkyl		hydroxamate	YES	1.86
5EF7	A	aromatic	YES	hydroxamate	YES	1.90
5EF7	B	aromatic	YES	hydroxamate	YES	1.90
5EF8	A	alkyl		hydroxamate		2.60
5EF8	B	alkyl		hydroxamate		2.60
5EFB	D	alkyl	YES	hydroxamate		2.54
5EFG	A					2.25
5EFG	D					2.25
5EFH	A	alkyl		trifluoroethyl-diol		2.16
5EFH	D	alkyl		trifluoroethyl-diol		2.16
5EFJ	D					1.73
5EFK	A					1.82
5EFK	B					1.82
5EFN	A					1.80
5EFN	B					1.80
5G0G	A	alkyl	YES	hydroxamate		1.50
5G0H	A	alkyl	YES	hydroxamate		1.60
5G0I	A	aromatic	YES	hydroxamate	YES	1.99
5G0I	B	aromatic	YES	hydroxamate	YES	1.99
5G0J	A					2.88
5W5K	A	aromatic	YES	hydroxamate	YES	2.70
5W5K	B	aromatic	YES	hydroxamate	YES	2.70
5W5K	C	aromatic	YES	hydroxamate	YES	2.70
5WGI	A	alkyl	YES	hydroxamate		1.05
5WGK	A	aromatic	YES	hydroxamate	YES	1.82
5WGL	A	alkyl	YES	hydroxamate		1.70
5WGL	B	alkyl	YES	hydroxamate		1.70
5WGM	A	aromatic	YES	hydroxamate	YES	1.75
5WGM	B	aromatic	YES	hydroxamate	YES	1.75
5WGM	C	aromatic	YES	hydroxamate	YES	1.75
6CGP	A	aromatic		hydroxamate	YES	2.50
6CSP	A	alkyl		hydroxamate		1.24
6CSP	B	alkyl		hydroxamate		1.24
6CSQ	A	alkyl		hydroxamate		2.03
6CSQ	B	alkyl		hydroxamate		2.03
6CSR	A	aromatic		hydroxamate	YES	1.62
6CSR	B	aromatic		hydroxamate	YES	1.62
6CSS	A	alkyl		hydroxamate		1.70
6CSS	B	alkyl		hydroxamate		1.70

6CW8	A	aromatic	YES	hydroxamate	YES	1.90
6CW8	B	aromatic	YES	hydroxamate	YES	1.90
6DVL	A	aromatic	YES	hydroxamate	YES	2.10
6DVL	B	aromatic	YES	hydroxamate	YES	2.10
6DVM	A	aromatic	YES	hydroxamate	YES	1.47
6DVM	B	aromatic	YES	hydroxamate	YES	1.47
6DVM	C	aromatic	YES	hydroxamate	YES	1.47
6DVM	D	aromatic	YES	hydroxamate	YES	1.47
6DVN	A	aromatic	YES	hydroxamate	YES	2.20
6DVN	B	aromatic	YES	hydroxamate	YES	2.20
6DVN	C	aromatic	YES	hydroxamate	YES	2.20
6DVN	D	aromatic	YES	hydroxamate	YES	2.20
6DVO	A	aromatic	YES	hydroxamate		1.98
6MR5	A	alkyl	YES	2-sulfanylacetamide		1.85
6MR5	B	alkyl	YES	2-sulfanylacetamide		1.85
6PYE	A	alkyl	YES	hydroxamate		1.48
6PYE	B	alkyl	YES	hydroxamate		1.48
6PZO	A	aromatic	YES	hydroxamate	YES	1.50
6PZO	B	aromatic		hydroxamate		1.50
6PZR	A	alkyl		hydroxamate		2.30
6PZR	B	alkyl		hydroxamate		2.30
6PZS	A	aromatic	YES	hydroxamate	YES	1.79
6PZU	A	aromatic	YES	hydroxamate		1.74
6PZU	B	aromatic	YES	hydroxamate		1.74
6Q0Z	A	heteroaromatic	YES	hydroxamate		1.75
6Q0Z	B	heteroaromatic		hydroxamate		1.75
6R0K	A	heteroaromatic	YES	hydroxamate		1.15

6TCY	A	aromatic	YES	hydroxamate		1.60
6TCY	B	aromatic	YES	hydroxamate		1.60
6THV	A	aromatic	YES	hydroxamate	YES	1.10
6V79	A	aromatic	YES	hydroxamate	YES	2.04
6V79	B	aromatic	YES	hydroxamate	YES	2.04
6V7A	A	aromatic	YES	hydroxamate		2.09
6VNR	A	aromatic	YES	hydroxamate	YES	1.94
6VNR	B	aromatic	YES	hydroxamate	YES	1.94

Note: [a] Crystal structure of human CDII HDAC6. All other crystal structures are from Danio Rerio CDII HDAC6.

A visual inspection of the aligned complexes allowed the identification of a set of conserved water molecules in proximity of the cap and ZBG regions in the HDAC6 catalytic site (Table A), which are often involved in the binding of the crystallographic ligands.
Particular attention was devoted to the crystal structure resolution, presence of water molecules near the cap and catalytic zinc regions, and on the chemical structure of co-crystallized ligands, during the selection process of HDAC6 crystal structures for the structure-based calculations. The 5WGI ultrahigh resolution crystal structure HDAC6, which is in complex with Trichostatin A, was selected as a suitable conformation for molecular docking calculations[12]. Indeed, 5WGI derived from crystallographic experiments on the catalytic domain II of *danio rerio* HDAC6. Therefore, it presents few differences on the amino acid composition, in proximity of the protein active site, with respect to the human HDAC6. Although these differences do not appear to affect ligand binding, we mutated the Asn530, Phe533, Glu638 and Asn645 residues in 5WGI (*danio rerio*) to Asp530, Tyr533, Asp638 and Met645 (*human*), respectively. Then, the computationally mutated complex was preprocessed with the *Protein Preparation Wizard* utility of the Schrödinger Suite 2020-1[13,14]. In particular, atom types and bond connectivity were checked, and missing residues side chains fixed. Afterwards, missing hydrogen atoms were added, and the resulting ligand-protein complex minimized. Ions, solvent and water molecules were finally removed from the prepared complex. His573 and His574 were defined as positively charged and neutral, respectively, in agreement with previous observations[12,15,16].
The 3-hydroxy-isoxazoles (see Figure 1 in the main section of the article) were first manually drawn in Maestro and prepared for the structure-based calculations by using the *LigPrep* utility[2]. In particular, protonation states and tautomers potentially present at physiological pH were generated. Moreover, additional metal binding states were also calculated. The 3-hydroxy-isoxazole moiety could exist in the keto and enol tautomeric forms at physiological pH, which might potentially establish different geometries of coordination with the catalytic Zn^{2+} in HDAC6. To evaluate the tautomeric preference of the 3-hydroxy-isoxazole chemical moiety in water solvent, quantum-mechanical calculations were performed by using Jaguar, with using default settings[17,18].
The enol tautomeric form resulted as the most probable for the 3-hydroxy-isoxazole according to quantum-mechanical calculations. Therefore, docking analyses were performed on compounds in their enol tautomeric form. Docking calculations were performed on the active site of the prepared receptor by using Glide[19,20]. The receptor grid was centered at the centroid of the bound Trichostatin A (TSN) ligand (outer box of 10 Å × 10 Å × 10 Å). Default settings were used for the grid generation, except for the scaling factor of the Van der Waals radii that was set to 1.0. The

docking protocol was validated by redocking the co-crystallized TSN ligand, obtaining satisfactory results (Root Mean Square Deviation value ≤ 2.0Å).
A visual inspection of the obtained poses was performed to help discussing the structure-activity relationships of the investigated series of ligands.

1.2 Synthetic Procedures

Materials and Instrumentations

All solvents, reagents and other chemicals were used as purchased without further purification, unless otherwise specified. Air or moisture sensitive reactions were performed under argon or nitrogen atmosphere. All the reactions were monitored using thin layer chromatography (TLC) on silica gel plates (60F-254, *Merck*) and the chromatograms were displayed by UV-light (254 nm), $KMnO_4$ alkaline aqueous solution, $FeCl_3$ 1% in ethanol or by a phenylhydrazine solution. All the reaction crude purifications were carried out with column liquid chromatography (LC), using Merck silica gel 60 (230-400 mesh, ASTM) or using the Isolera One Flash Chromatography Instrument (*Biotage*). All the obtained products were structurally characterized by nuclear magnetic resonance (NMR) and mass spectrometry experiments that were carried out at the C.I.G.S - UNIMORE. ^1H and ^{13}C NMR spectra (1D and 2D experiments) were recorded on a *DPX-400 Avance* spectrometer (*Bruker*) at 400 MHz. Chemical shifts are expressed in ppm (δ) and calibrated on the signal of the solvent ($CDCl_3$ δ 77.04, DMSO-d6 δ 39.5, $(CD_3)_2CO$ δ 29.84 and 206.26 for ^{13}C; $CDCl_3$ δ 7.26, DMSO-d6 δ 2.50, $(CD_3)_2CO$ δ 2.05 for ^1H). The NMR data were reported as follows: chemical shift, multiplicity (s, singlet; d, doublet; t, triplet; q, quartet; qnt, quintet; sxt, sextet; m, multiplet; br, broad), number of proton/carbons, coupling constant (Hz) and peak assignment (benzyl (Bn), furan (Fur), isoxazole (Isox), naphthyl (Naph), phenyl (Ph), pyridinyl (Pyr), thiophene (Thio)). ^1H-^{13}C heteronuclear multiple quantum coherence (HMQC) and heteronuclear multiple bond connectivity (HMBC) experiments were recorded for determination of ^1H-^{13}C correlations. The purity degree of all synthesized compounds was considered good after NMR analysis (> 90%). As an example, the ^1H NMR spectra of **13**, **17**, **23**, **25** and **27** are reported below in this section (see pages S33-S35). The mass analyses were performed using the UHPLC-MS Q Exactive instrument (*Thermo Scientific*) equipped with a *UHPLC Ultimate 3000* system coupled to a *Q Exactive™ Hybrid Quadrupole-Orbitrap™* mass spectrometer. The melting points were determined with a Stuart SMP3 apparatus and are uncorrected. The solvent names have been abbreviated as follows: ethyl acetate (EtOAc), dimethylsulfoxide (DMSO), dichloromethane (DCM), cyclohexane (CE), diethyl ether (Et_2O), methanol (MeOH), ethanol (EtOH), tetrahydrofuran (THF), dimethylformamide (DMF).

5-phenylisoxazol-3-ol **13**

It was obtained during the synthesis of the N-hydroxy-3-phenyl-propiolamide **12**, as described in the main manuscript. The compound is a pale orange solid; m.p. = 174-176°C

^1H-NMR (DMSO-d_6, 400 MHz): δ 6.54 (s, 1H, CH-4 Isox); 7.46-7.52 (m, 3H, CH-3, CH-4, CH-5 Ph); 7.79 (dd, 2H, J = 1.85, 7.99 Hz, CH-2, CH-6 Ph); 11.33 (s, 1H, OH).

^{13}C-NMR (DMSO-d_6, 400 MHz): δ 99 (C-4 Isox); 127 (C-2, C-6 Ph); 129 (C-1 Ph); 129.7 (C-4 Ph); 131 (C-4, C5 Ph); 167 (C-5 Isox); 169 (C-3 Isox).

HRMS m/z $[M+H]^+$ calcd. for $C_9H_8NO_2^+$: 162.0550; found: 162.0548.

Pathway A

The 3-hydroxy-5-arylisoxazoles **14-16** were synthesized respectively by direct cyclization of the 2,3-dibromo-3-arylpropanoate esters **14.1-16.1** with hydroxylamine hydrochloride, in ethanol solution of NaOH, at reflux. **14.1-16.1** were obtained from the corresponding cinnamic esters **14.2-16.2** using bromine in DCM at room temperature. The cinnamic esters **14.2** and **15.2** were prepared from the corresponding commercially available cinnamic acids A and B using thionyl chloride ($SOCl_2$) in ethanol, at reflux. The cinnamic acid C was treated with dimethyl sulfate ($(CH_3)_2SO_4$) and potassium carbonate (K_2CO_3), in acetone, at reflux. In this way the hydroxy group in *para* position was also methylated giving the ester **16.2**. However, following this first pathway (**Scheme S1**), the overall final yields were not satisfactory, therefore a second synthetic approach was adopted (Pathway B).

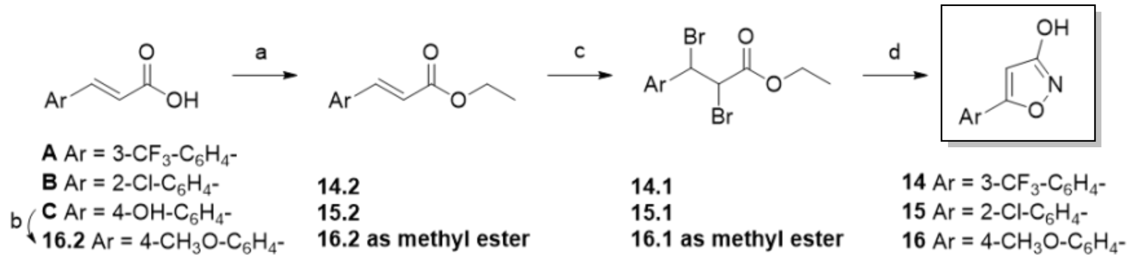

Scheme S1. Reagents and conditions: **(a)** $SOCl_2$ (ex.), EtOH (20 mL); 0°C to reflux, 2h, 92% yield. **(b)** $(CH_3)_2SO_4$ (2 eq.), K_2CO_3 (2.5 eq.), Acetone (15 mL); reflux, 3h, 85% yield. **(c)** Br_2 (1.1 eq.), DCM (15 mL); 0°C to r.t., 2h, 89% yield. **(d)** NH_2OH • HCl (1,5 eq.), NaOH (3.5 eq.), EtOH (20 mL); reflux, 3h, 33% yield.

Pathway B

The 3-hydroxy-5-arylisoxazoles **17-22** and **23.1** were synthesized starting from the corresponding 3-arylpropiolate esters **17.1-22.1** and **23.2**. In particular, to obtain the 3-hydroxy-5-arylisoxazoles **17** and **18** we started, as in Pathway A, from the commercially available cinnamic acids D and E that were esterified and subsequently brominated, obtaining **17.3** and **18.3**. In the case of the ethyl 3-(4-hydroxyphenyl)acrylate **17.5**, a NS reaction with bromomethylbenzene was performed before bromination in order to obtain the benzyloxy derivative **17.4**. Then, all the 2,3-dibromo-3-arylpropanoate esters were transformed into the 3-arylpropiolic acids by dehydrohalogenation reaction with KOH in ethanol, at reflux. After, a re-esterification with $(CH_3)_2SO_4$ was run. Finally, the 3-arylpropiolic esters **17.1** and **18.1** were treated with aqueous hydroxylamine in a methanol solution of NaOH, at room temperature, to give the final products **17** and **18**. Also **19-22** and **23.1** were obtained in the same way. The starting cinnamic esters **19.4-22.4** and **23.5** were brominated, then dehydrohalogenation and the subsequent re-esterification were performed. Finally, the cyclization reaction followed. The above listed cinnamic esters were prepared by Wittig reaction between the appropriate arylaldehydes (G, H, I, L, M, N) and the phosphonium salt **30.2**, which was obtained by reacting the methyl 2-bromoacetate (F) with triphenylphosphine ($P(Ph)_3$), in toluene, at reflux (**Scheme S2**). In this pathway, unlike the previous one, a different procedure was adopted for the final cyclization reaction. In fact, aqueous hydroxylamine was used instead of hydroxylamine hydrochloride. From here this last reaction was always carried out in this way. As last variation it was heated for the first time at 70°C to obtain **22**. During the mild heating it was observed by TLC that the balance between the hydroxamic acid and the isoxazole form shifted towards the latter. This allowed us to achieve higher yields. Therefore, the subsequent cyclization reactions were all carried out at 70°C. At the end of this pathway the final compound **23** was obtained from a Buchwald-Hartwig amination of the 5-(4-bromophenyl)isoxazole **23.1**.

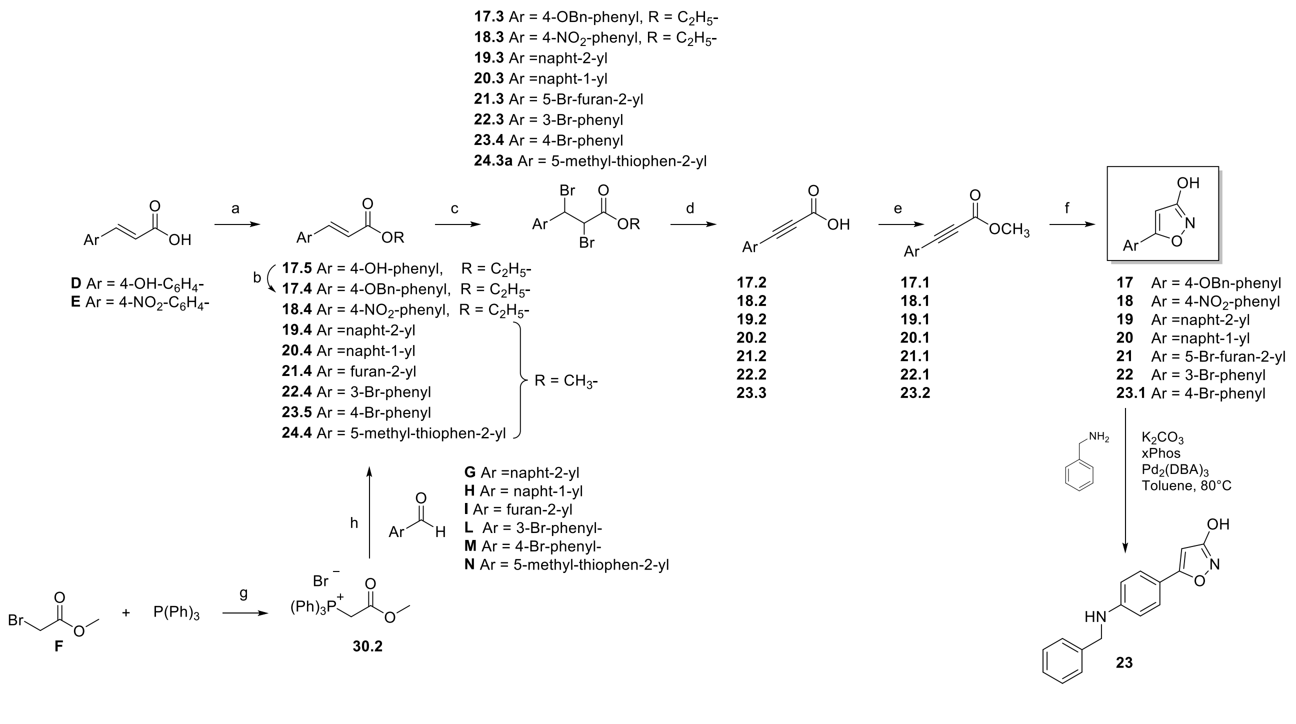

Scheme S2. Reagents and conditions: **(a)** $SOCl_2$ (ex.), EtOH (20 mL); 0°C to reflux, 2h, 92% yield. **(b)** bromomethylbenzene (1.1 eq.), K_2CO_3 (1.5 eq.), DMF (3 mL); 60°C, 3h, 72% yield. **(c)** Br_2 (1.1 eq.), DCM (15 mL); 0°C to r.t., 2h, 89% yield. **(d)** KOH (8 eq.), EtOH (20 mL); reflux, 2h, 3-30% yield. **(e)** $(CH_3)_2SO_4$ (1 eq.), K_2CO_3 (1.5 eq.), Acetone (15 mL); reflux, 1h, 32% yield. **(f)** aq. NH_2OH 50% (1 mL x 0.35 mmol ester), NaOH (8 eq.),

MeOH (= mL of NH_2OH); r.t. or 70°C, 3h, 43% yield. **(g)** methyl 2-bromoacetate, $P(Ph)_3$ (1.1 eq.), Toluene (40 mL); reflux, 7h, quant. yield. **(h)** arylaldeyde, (2-methoxy-2-oxoethyl)triphenylphosphonium bromide (1.2 eq.), K_2CO_3 0.1 M (10 mL x mmol of R-$P(Ph)_3Br$); reflux, 3h, 45% yield.

Starting from the methyl 3-arylpropiolates this synthetic pathway gave the highest yields. However, many byproducts were still present, consequently another synthetic route was tried to obtain the alkynes in a cleaner way. Both sodium hydride (NaH) in anhydrous toluene and potassium *tert*-butoxide (*t*-ButOK) in anhydrous THF were used but each attempt failed. Finally, starting from the mono-brominated byproduct **24.3b**, the methyl 2-bromo-3-(5-methylthiophen-2-yl)acrylate obtained during the bromination of **24.4** (**Scheme S3**), a new way to synthesize the methyl 3-arylpropiolates was adopted. Thus, Pathway C took place.

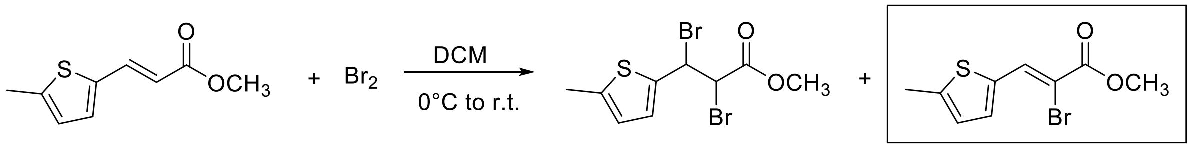

24.4 **24.3a** **24.3b**

Scheme S3

Pathway C

The 3-hydroxy-5-arylisoxazoles **24-27** were synthesized as in Pathway B starting from the methyl 3-arylpropiolates **24.1-27.1** that were cyclized with aqueous hydroxylamine. These esters were produced from the 3-arylpropiolic acids **24.2-27.2** that were obtained from the corresponding α-bromoacrylates. Following the procedure reported by Kim et al.[22], the α-bromoacrylates **24.3b** and **25.3-27.3** were reacted with sodium amide ($NaNH_2$) and *t*-ButOK in anhydrous THF, at room temperature, giving the desired acids with good yields. As just described, the α-bromoacrylate **24.3b** was isolated from the bromination reaction in Pathway B (**Scheme S3**), the others (**25.3-27.3**) were synthesized according to Augustine et al.[23] The respective aldehydes O, P, Q were reacted with methyl 2-bromoacetate in the presence of titanium tetrachloride ($TiCl_4$) and triethylamine (TEA), in anhydrous DCM, under nitrogen flow and at room temperature (**Scheme S4**). The hypothesized mechanism, by which this selective (*Z*)-olefination takes place, involved a well-stabilized, six-membered, *Zimmerman-Traxler transition state*[23].

24.3b Ar = 5-methyl-thiophen-2-yl
25.3 Ar = piperon-2-yl
26.3 Ar = furan-2-yl
27.3 Ar = 4-(3,4-dicloro-benzyloxy)phenyl

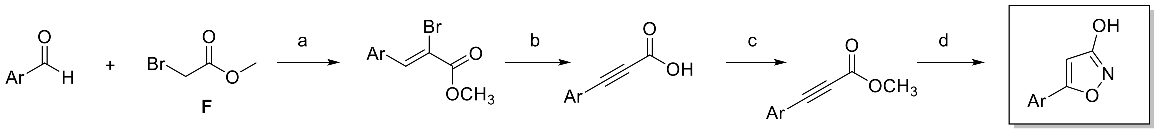

F

O Ar = piperon-2-yl
P Ar = furan-2-yl
Q Ar = 4-(3,4-dicloro-benzyloxy)phenyl

24.2
25.2
26.2
27.2

24.1
25.1
26.1
27.1

24 Ar = 5-methyl-thiophen-2-yl
25 Ar = piperon-2-yl
26 Ar = furan-2-yl
27 Ar = 4-(3,4-dicloro-benzyloxy)phenyl

Scheme S4. Reagents and conditions: **(a)** arylaldeyde, methyl 2-bromoacetate (1 eq.), $TiCl_4$ (1.2 eq.), TEA (2 eq.), anhydrous DCM (10 mL); N_2, r.t., 1h, 69% yield. **(b)** $NaNH_2$ (1.5 eq.), t-ButOK (1.5 eq.), anhydrous THF (10 mL); r.t., 1h, 95% yield. **(c)** $(CH_3)_2SO_4$ (1 eq.), K_2CO_3 (1.5 eq.), Acetone (15 mL); reflux, 1h, quant. yield. **(d)** aq. NH_2OH 50% (1 mL x 0.35 mmol ester), NaOH (8 eq.), MeOH : THF (= mL of NH_2OH); 60°C, 3h, 86% yield.

Pathway D

The only observed limitation of the previous synthetic procedure was its inefficacy when the starting 5-bromo-pyridin-2-yl carboxaldehyde (R) was used. Therefore, for the synthesis of the methyl 2-bromo-3-(5-bromo-pyridin-2-yl)acrylate **28.3**, a variant of the Wittig reaction was adopted[24]. The pyridinyl-carboxaldehyde above mentioned was treated with the brominated triphenylphosphonium ylide **30** and TEA, in DCM, at room temperature. **28.3** was then dehydrohalogenated to **28.2**, re-esterified to **28.1** that was cyclized to give the final **28**. The ylide **30** was previously prepared brominating the corresponding triphenylphosphonium ylide **30.1**. The triphenylphosphonium salt **30.2** produced immediately **30.1** using NaOH 1M in a mixture of DCM: H_2O 1:1, at room temperature (**Scheme S5**).

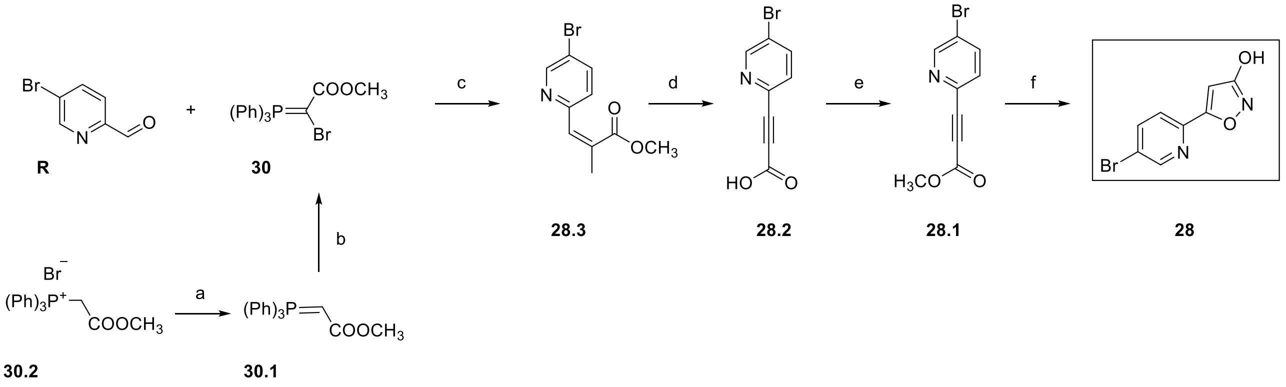

Scheme S5. Reagents and conditions: **(a)** NaOH 1M (1.1 eq.), DCM : H_2O 1:1 (40 mL); r.t., few min., 97% yield. **(b)** Br_2 (1.1 eq.), DCM (13 mL); r.t., 2h, quant. yield. **(c)** Aldehyde (0.66 eq.), TEA (5.28 eq.), DCM (9 mL), from 0°C to r.t., overnight, quant. yield. **(d)** $NaNH_2$ (1.5 eq.), t-ButOK (1.5 eq.), anhydrous THF (10 mL); r.t., 1h, 95% yield. **(e)** $(CH_3)_2SO_4$ (1 eq.), K_2CO_3 (1.5 eq.), acetone (15 mL); reflux, 1h, quant. yield. **(f)** aq. NH_2OH 50% (1 mL x 0.35 mmol ester), NaOH (8 eq.), CH_3OH : THF (= mL of NH_2OH); 60°C, 3h, 86% yield.

Pathway E

This last synthetic approach was used to obtain the 5-phenylisoxazole-3-carboxylic acid **29**. The ethyl 2,4-dioxo-4-phenylbutanoate (S) was cyclized with hydroxylamine (NH_2OH) hydrochloride in ethanol, at reflux, for 2 hours. The obtained ethyl 5-phenylisoxazole-3-carboxylate **29.1** was then hydrolyzed with NaOH 1M to give the desired final acid derivative (**Scheme S6**).

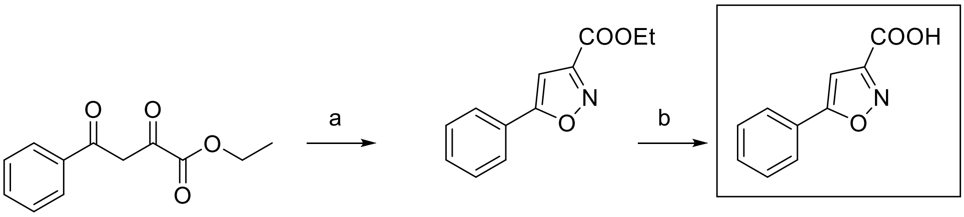

S **29.1** **29**

Scheme S6. Reagents and conditions: **(a)** NH_2OH • HCl (2.5 eq.), EtOH; reflux, 2h, quant. yield. **(b)** NaOH 1M, EtOH; r.t., 70% yield.

Synthesis of *methyl (E)- and (Z)-3-(4-methoxyphenyl)acrylate* 16.2 (path. A)

To a solution of 3-(4-hydroxyphenyl)acrylic acid (C) in 15 mL of acetone, K_2CO_3 (2.5 eq.) and $(CH_3)_2SO_4$ (2 eq.) were added. The reaction mixture was refluxed and stirred for three hours. Then the mixture was filtered and the solvent was concentrated under *vacuum*. A Na_2SO_4 saturated aqueous solution was added and this phase was extracted with Et_2O. The organic phase was washed with a NaCl saturated solution, dried over anhydrous Na_2SO_4, filtered and the solvent was concentrated under *vacuum*.

TLC CE : EtOAc 6:4, Rf: 0.68

Yield: 0.492 g (85%) of a white solid that was used without further purification.

^{1}H-NMR ($CDCl_3$, 400 MHz): δ 3.79 (s, 3H, -OCH_3); 3.84 (s, 3H, CH_3O-Ph); 6.30 (d, 1H, *J* = 16.05 Hz, CHC=O); 6.90 (dd, 2H, *J* = 1.94, 6.87 Hz, CH-3, CH-5 Ph); 7.47 (dd, 2H, *J* = 1.94, 6.87 Hz, CH-2, CH-6 Ph); 7.65 (d, 1H, *J* = 16.05 Hz, Ph-CH).

General procedure for the synthesis of *ethyl acrylate derivatives*

The appropriate cinnamic acid (1 eq.) was suspended in 20 mL of EtOH and the mixture was placed in an ice bath. Thionyl chloride was added dropwise to the suspension. The reaction mixture was stirred and refluxed for two hours. After it was cooled to room temperature and the solvent was evaporated under *vacuum*. The pH of the mixture was turned to basic with a saturated Na_2CO_3 solution. Then the aqueous phase was extracted with Et_2O. The organic phase was washed with brine, dried over anhydrous Na_2SO_4 and the solvent was concentrated under *vacuum*.

***ethyl (E)- and (Z)-3-(3-(trifluoromethyl)phenyl)acrylate* 14.2 (path. A)**

TLC CE : EtOAc 6:4, Rf: 0.76

Yield: 0.492 g (96%) of a solid that was used without further purification.

^{1}H-NMR ($CDCl_3$, 400 MHz): δ 1.35 (t, 3H, *J* = 7.12 Hz, $C\underline{H}_3CH_2O$); 4.28 (q, 2H, *J* = 7.12 Hz, $CH_3C\underline{H}_2O$); 6.49 (d, 1H, *J* = 16.04 Hz, CHC=O); 7.52 (t, 1H, *J* = 7.87 Hz, CH-5 Ph); 7.62 (d, 1H, *J* = 7.87 Hz, CH-6 Ph); 7.67-7.71 (m, 2H, Ph-C$\underline{H}$, CH-4 Ph); 7.76 (s, 1H, CH-2 Ph).

***ethyl (E)- and (Z)-3-(2-chlorophenyl)acrylate* 15.2 (path. A)**

TLC CE : EtOAc 6:4, Rf: 0.71

Yield: 0.550 g (95%) of a solid that was used without further purification.

^{1}H-NMR ($CDCl_3$, 400 MHz): δ 1.35 (t, 3H, *J* = 7.14 Hz, $C\underline{H}_3CH_2O$); 4.28 (q, 2H, J = 7.14 Hz; $CH_3C\underline{H}_2O$); 6.43 (d, 1H, *J* = 16.18 Hz, CHC=O); 7.27-7.32 (m, 2H, CH-4, CH-5 Ph); 7.41 (dd, 1H, *J* = 1.82, 7.79 Hz, CH-6 Ph); 7.62 (dd, 1H, *J* = 2.08, 7.20 Hz, CH-3 Ph); 8.08 (d, 1H, *J* = 16.18 Hz, Ph-C$\underline{H}$).

***ethyl (E)- and (Z)-3-(4-nitrophenyl)acrylate* 18.4 (path. B)**

TLC CE : EtOAc 6:4, Rf: 0.70

Yield: 0.535 g (93%) of a pale-yellow solid that was used without further purification.

^{1}H-NMR ($CDCl_3$, 400 MHz): δ 1.36 (t, 3H, *J* = 7.12 Hz, $C\underline{H}_3CH_2O$); 4.20 (q, 2H, *J* = 7.12 Hz, $CH_3C\underline{H}_2O$); 6.60 (d, 1H, *J* = 16.04 Hz, CHC=O); 7.26 (d, 1H, *J* = 16.04 Hz, Ph-C$\underline{H}$); 8.03 (d, 2H, *J* = 7.97 Hz, CH-2, CH-6 Ph); 8.21 (d, 2H, *J* = 7.97 Hz, CH-3, CH-5 Ph).

***ethyl (E)- and (Z)-3-(4-hydroxyphenyl)acrylate* 17.5 (path. B)**

TLC CE : EtOAc 6:4, Rf: 0.48

Silica gel column chromatography with CE : EtOAc 7:3 elution. The title compound was obtained as a solid. Yield: 0.498 g (43%).

^{1}H-NMR ($CDCl_3$, 400 MHz): δ 1.33 (t, 3H, *J* = 7.15 Hz, $C\underline{H}_3CH_2O$); 4.25 (q, 2H, *J* = 7.15 Hz, $CH_3C\underline{H}_2O$); 5.63 (s, 1H, Ph-O$\underline{H}$); 6.29 (d, 1H, *J* = 16.08 Hz, CHC=O); 6.85 (d, 2H, *J* = 8.65 Hz, CH-3, CH-5 Ph); 7.42 (d, 2H, *J* = 8.65 Hz, CH-2, CH-6 Ph); 7.62 (d, 1H, *J* = 16.08 Hz, Ph-C$\underline{H}$).

Synthesis of *ethyl (E)- and (Z)-3-(4-benzyloxy)phenyl)acrylate* 17.4 (path. B)
Bromomethyl-benzene (1.1 eq.) and K_2CO_3 (1.5 eq.) were added to a solution of 3-(4-hydroxyphenyl)acrylic acid **17.5** in 3 mL of DMF. The reaction mixture was stirred and heated at 60°C for three hours. Then it was diluted with water and extracted with Et_2O. The organic phase was washed with a saturated solution of NaCl, dried over anhydrous Na_2SO_4, filtered and the solvent was concentrated under *vacuum*.
TLC CE : EtOAc 6:4, Rf: 0.75
Yield: 0.653 g (93%) of a yellow solid that was used without further purification.
^{1}H-NMR ($CDCl_3$, 400 MHz): δ 1.33 (t, 3H, J = 7.22 Hz, C$\underline{H}_3$$CH_2O$); 4.25 (q, 2H, J = 7.22 Hz, CH_3C$\underline{H}_2$O); 5.09 (s, 2H, Ph-C$\underline{H}_2$O); 6.30 (d, 1H, J = 16.06 Hz, CHC=O); 6.97 (dd, 2H, J = 1.83, 6.81 Hz, CH-3, CH-5 Ph); 7.33-7.44 (m, 5H, CH Bn); 7.47 (dd, 2H, J = 1.83, 6.81 Hz, CH-2, CH-6 Ph); 7.63 (d, 1H, J = 16.06 Hz, Ph-C$\underline{H}$).

Synthesis of *(2-methoxy-2-oxoethyl)triphenylphosphonium bromide* 30.2 (path. B)
Methyl 2-bromoacetate (F) was added to a suspension of triphenylphosphine (1.1 eq.) in 20 mL of toluene. The reaction was stirred and refluxed for seven hours. Then the suspension was filtered and the solid was left to dry overnight.
Yield: 8.84 g (96%) of a white solid that was used without further purification.
^{1}H-NMR (DMSO, 400 MHz): δ 3.61 (s, 3H, OCH_3); 5.36 (d, 2H, J = 14.4 Hz, C$\underline{H}_2$-P(Ph)$_3$); 7.76-7.83 (m, 12H, CH Ph); 7.88-7.93 (m, 3H, CH Ph).

General procedure for the synthesis of *methyl acrylate derivatives* (Wittig reaction)
The appropriate aldehyde (1.5 eq.) was added to a suspension of (2-methoxy-2-oxoethyl)triphenylphosphonium bromide in a K_2CO_3 0.1 M solution (10 mL x mmol of R-P(Ph)$_3$Br). The reaction mixture was stirred and refluxed for 3 hours. Then it was extracted with EtOAc and the organic phase was washed with brine saturated solution, dried over anhydrous Na_2SO_4, filtered and the solvent was concentrated under *vacuum*.

***methyl (E)- and (Z)-3-(naphthalene-2-yl)acrylate* 19.4 (path. B)**
TLC CE : EtOAc 8:2, Rf: 0.66
The starting aldehyde was still present in the crude that was purified by reaction with dinitrophenylhydrazine in EtOH, at reflux. The suspension was filtered and the filtrate concentrated under *vacuum*.
Silica gel column chromatography with CE : EtOAc 8:2 elution.
Yield: 0.498 g (98%) of a white solid.
^{1}H-NMR ($CDCl_3$, 400 MHz): δ 3.8 (s, 3H, OCH_3); 6.55 (d, 1H, J = 16.09 Hz, CHC=O); 7.50-7.52 (m, 2H, CH-6, CH-7 Naph); 7.66 (dd, 1H, J = 1.58, 8.61 Hz, CH-3 Naph); 7.82-7.87 (m, 4H, CHC=C, CH-4, CH-5, CH-8 Naph); 7.93 (d, 1H, J = 1.50, CH-1 Naph).

***methyl (E)- and (Z)-3-(naphthalene-1-yl)acrylate* 20.4 (path. B)**
TLC CE : EtOAc 8:2, Rf: 0.73
Silica gel column chromatography with CE : EtOAc 8:2 elution.
Yield: 0.302 g (45%) of a pale yellow solid.
^{1}H-NMR ($CDCl_3$, 400 MHz): δ 3.85 (s, 3H, OCH_3); 6.53 (d, 1H, J = 15.77 Hz, CHC=O); 7.47-7.58 (m, 3H, CH-3, CH-6, CH-7 Naph); 7.75 (d, 1H, J = 7.25 Hz, CH-2 Naph); 7.89 (t, 2H, J = 8.07 Hz, CH-5, CH-8 Naph); 8.29 (d, 1H, J = 8.36 Hz CH-4 Naph); 8.53 (d, 1H, J = 15.77 Hz, Naph-C$\underline{H}$).

***methyl (E)- and (Z)-3-(furan-2yl)acrylate* 21.4 (path. B)**
TLC CE : EtOAc 8:2, Rf: 0.38
Silica gel column chromatography with CE : EtOAc 8:2 elution.
Yield: 0.280 g (35%) of a light brown solid.

^{1}H-NMR ($CDCl_3$, 400 MHz): δ 3.78 (s, 3H, OCH_3); 6.31 (d, 1H, J = 15.93 Hz, CHC=O); 6.46 (dd, 1H, J = 1.87, 3.38 Hz, CH-4 Fur); 6.60 (d, 1H, J = 3.40 Hz, CH-3 Fur); 7.43 (d, 1H, J = 15.93 Hz, Fur-C$\underline{H}$); 7.47 (d, 1H, J = 1.38 Hz, CH-5 Fur).

***methyl (E)- and (Z)-3-(3-bromophenyl)acrylate* 22.4 (path. B)**
TLC CE : EtOAc 8:2, Rf: 0.58
Silica gel column flash chromatography with CE : EtOAc 8:2 elution.
Yield: 0.499 g (50%) of a yellow crystalline solid.
^{1}H-NMR ($CDCl_3$, 400 MHz): δ 3.81 (s, 3H, OCH_3); 6.43 (d, 1H, J = 15.98 Hz, CHC=O); 7.25 (t, 1H, J = 7.81 Hz, CH-5 Ph); 7.43 (d, 1H, J = 7.71 Hz, CH-4 Ph); 7.50 (ddd, 1H, J = 1.72, 2.79, 8.05 Hz, CH-6 Ph); 7.60 (d, 1H, J = 15.98 Hz, Ph-C$\underline{H}$); 7.66 (t, 1H, J = 1.72 Hz, CH-2 Ph).

***methyl (E)-3-(4-bromophenyl)acrylate* 23.5 (path. B)**
TLC CE : EtOAc 9:1, Rf: 0.58
Automated flash chromatography (*Isolera-Biotage* system) with CE : EtOAc (7 → 20% EtOAc) elution.
Yield: 0.72 g (96%) of a white solid.
^{1}H-NMR ($CDCl_3$, 400 MHz): δ 3.81 (s, 3H, OCH_3); 6.42 (d, 1H, J = 15.95 Hz, CHC=O); 7.38 (d, 2H, J = 8.54 Hz, CH-3, CH-5 Ph); 7.52 (d, 2H, J = 8.61 Hz, CH-2, CH-6 Ph); 7.62 (d, 1H, J = 16.04 Hz, Ph-C$\underline{H}$).

***methyl (E)- and (Z)-3-(5-methyltiophen-2-yl)acrylate* 24.4 (path. B)**
TLC CE : EtOAc 8:2, Rf: 0.63
Silica gel column chromatography with CE : EtOAc 8:2 elution.
Yield: 0.379 g (76%) of a yellow solid.
^{1}H-NMR ($CDCl_3$, 400 MHz): δ 2.49 (s, 3H, CH_3-Thio); 3.77 (s, 3H, OCH_3); 6.1 (d, 1H, J = 15.73 Hz, CHC=O); 6.7 (d, 1H, J = 3.43 Hz, CH-3 Thio); 7.05 (d, 1H, J = 3.43 Hz, CH-4 Thio); 7.69 (d, 1H, J = 15.73 Hz, Thio-C$\underline{H}$).

General procedure for the synthesis of *dibromo-derivatives of cinnamic acid*
The appropriate 3-arylacrylate, methyl or ethyl ester, was solubilized in 5 ml of DCM. Then a solution of bromine (Br_2; 1.1 eq.) in 10 ml of DCM was added dropwise at 0°C. The reaction mixture was stirred at room temperature for two hours. After the solvent was concentrated under *vacuum* to eliminate also the possible excess of Br_2.

***ethyl 2,3-dibromo-3-(3-(trifluoromethyl)phenyl)propanoate* 14.1 (path. A)**
TLC CE : EtOAc 8:2, Rf: 0.48
Yield: 0.814 g (quant.) of a viscous liquid that was used without further purification.
^{1}H-NMR ($CDCl_3$, 400 MHz): δ 1.36 (t, 3H, J = 7.12 Hz, C$\underline{H}_3$$CH_2$O); 4.31 (q, 2H, J = 7.12 Hz, CH_3C$\underline{H}_2$O); 4.82 (d, 1H, J = 11.8 Hz, Br-CHC=O); 5.45 (d, 1H, J = 11.8, Ph-C$\underline{H}$Br); 7.52 (t, 1H, J = 7.77 Hz, CH-5 Ph); 7.62 (d, 1H, J = 7.97 Hz, CH-6 Ph); 7.69 (d, 1H, J = 7.77 Hz, CH-4 Ph); 7.76 (s, 1H, CH-2 Ph).

***ethyl 2,3-dibromo-3-(2-(chlorophenyl)propanoate* 15.1 (path. A)**
TLC CE : EtOAc 8:2, Rf: 0.73
Yield: 0.932 g (97%) of a viscous liquid that was used without further purification.
^{1}H-NMR ($CDCl_3$, 400 MHz): δ 1.39 (t, 3H, J = 7.12 Hz, C$\underline{H}_3$$CH_2$O); 4.37 (q, 2H, J = 7.12 Hz, CH_3C$\underline{H}_2$O); 4.91 (d, 1H, J = 15.0 Hz, Br-CHC=O); 5.92 (d, 1H, J = 15.0 Hz, Ph-C$\underline{H}$-Br); 7.28-7.34 (m, 2H, CH-4, CH-5 Ph); 7.41 (dd, 1H, J = 1.67, 7.74 Hz, CH-6 Ph); 7.48 (d, 1H, J = 7.8 Hz, CH-3 Ph).

***methyl 2,3-dibromo-3-(4-methoxyphenyl)propanoate* 16.1 (path. A)**
TLC CE : EtOAc 8:2, Rf: 0.39
Yield: 0.892 g (91%) of a pink solid that was used without further purification.
^{1}H-NMR ($CDCl_3$, 400 MHz): δ 3.82 (s, 3H, OCH_3); 3.89 (s, 3H, C<u>H</u>$_3$O-Ph); 4.83 (d, 1H, *J* = 11.28 Hz, Br-CHC=O); 5.34 (d, 1H, *J* = 11.28 Hz, Ph-C<u>H</u>-Br); 6.90 (d, 2H, *J* = 8.80 Hz, CH-3, CH-5 Ph); 7.33 (d, 2H, *J* = 8.80 Hz, CH-2, CH-6 Ph).

***ethyl 3-(4-(benzyloxy)phenyl)-2,3-dibromopropanoate* 17.3 (path. B)**
TLC CE : EtOAc 8:2, Rf: 0.72
Yield: 1.07 g (89%) of a pale-yellow solid that was used without further purification.
^{1}H-NMR ($CDCl_3$, 400 MHz): δ 1.37 (t, 3H, *J* = 7.12 Hz, C<u>H</u>$_3$CH$_2$O); 4.35 (q, 2H, *J* = 7.12 Hz, CH$_3$C<u>H</u>$_2$O); 4.80 (d, 1H, *J* = 11.77 Hz, Br-CHC=O); 5.07 (s, 2H, PhC<u>H</u>$_2$O); 5.35 (d, 1H, *J* = 11.77, Ph-C<u>H</u>-Br); 6.98 (d, 2H, *J* = 8.72 Hz, CH-3, CH-5 Ph); 7.32-7.42 (m, 7H, CH-2 Ph, CH-6 Ph, CH Bn).

***ethyl 2,3-dibromo-3-(4-nitrophenyl)propanoate* 18.3 (path. B)**
TLC CE : EtOAc 8:2, Rf: 0.52
Yield: 0.365 g (96%) of a viscous liquid that was used without further purification.
^{1}H-NMR ($CDCl_3$, 400 MHz): δ 1.38 (t, 3H, *J* = 8.0 Hz, C<u>H</u>$_3$CH$_2$O); 4,37 (q, 2H, *J* = 8.0 Hz, CH$_3$C<u>H</u>$_2$O); 4.77 (d, 1H, *J* = 11.8 Hz, Br-CHC=O); 5.38 (d, 1H, *J* = 11.8 Hz, Ph-C<u>H</u>-Br); 7.59 (dd, 2H, *J* = 2.17, 6.90 Hz, CH-2, CH-6 Ph); 8.26 (d, 2H, *J* = 2.17, 6.90 Hz, CH-3, CH-5 Ph).

***methyl 2,3-dibromo-3-(naphthalen-2-yl)propanoate* 19.3 (path. B)**
TLC CE : EtOAc 8:2, Rf: 0.62
Yield: 0.809 g (93%) of an orange solid that was used without further purification.
^{1}H-NMR ($CDCl_3$, 400 MHz): δ 3.86 (s, 3H, OCH_3); 4.90 (d, 1H, *J* = 11.28 Hz, Br-CHC=O); 5.41 (d, 1H, *J* = 11.28 Hz, Ph-C<u>H</u>-Br); 7.50-7.52 (m, 2H, CH-6, CH-7 Naph); 7.66 (dd, 1H, *J* = 1.58, 8.61 Hz, CH-3 Naph); 7.81-7.89 (m, 3H, CH-4, CH-5, CH-8 Naph); 7.93 (s, 1H, CH-1 Naph).

***methyl 2,3-dibromo-3-(naphthalen-1-yl)propanoate* 20.3 (path. B)**
TLC CE : EtOAc 8:2, Rf: 0.60
Yield: 0.460 g (92%) of an orange oil that was used without further purification.
^{1}H-NMR ($CDCl_3$, 400 MHz): δ 3.96 (s, 3H, OCH_3); 5.15 (d, 1H, *J* = 11.64 Hz, Br-CH-C=O); 6.29 (d, 1H, *J* = 11.64 Hz, Ph-C<u>H</u>-Br); 7.52-7.57 (m, 2H, CH-3, CH-7 Naph); 7.63-7.70 (m, 2H, CH-2, CH-6 Naph); 7.89-7.92 (m, 2H, CH-5, CH-8 Naph); 8.16 (d, 1H, *J* = 8.26 Hz, CH-4 Naph).

***methyl 2,3-dibromo-3-(5-bromofuran-2-yl)propanoate* 21.3 (path. B)**
TLC CE : EtOAc 8:2, Rf: 0.71
Yield: 0.314 g (28%) of a dark green oil that was used without further purification.
^{1}H-NMR ($CDCl_3$, 400 MHz): δ 3.88 (s, 3H, CH_3O); 4.91 (d, 1H, *J* = 11.63 Hz, Br-CH-C=O); 5.40 (d, 1H, *J* = 11.62 Hz, Fur-C<u>H</u>-Br); 6.31 (d, 1H, *J*= 3.51 Hz, CH-3 Fur); 6.45 (d, 1H, *J* = 3.51 Hz, CH-4 Fur).

***methyl 2,3-dibromo-3-(3-bromophenyl)propanoate* 22.3 (path. B)**
TLC DCM : MeOH 9:1, Rf: 0.52
Yield: 0.796 g (96%) of an orange solid that was used without further purification.
^{1}H-NMR ($CDCl_3$, 400 MHz): δ 4.76 (d, 1H, J = 11.75 Hz, CHC=O); 5.25 (d, 1H, *J* = 11.75 Hz, Ph-CH-); 7.26 (t, 1H, *J* = 7.86 Hz, CH-5 Ph); 7.33 (dt, 1H, *J* = 1.27, 7.86 Hz, CH-6 Ph); 7.48 (dt, 1H, *J* = 1.41, 7.86 Hz, CH-4 Ph); 7.55 (t, 1H, *J* = 1.73 Hz, CH-2 Ph).

***methyl 2,3-dibromo-3-(4-bromophenyl)*propanoate 23.4 (path. B)**

TLC CE : EtOAc 9:1, Rf: 0.62
Yield: 0.960 g (86%) of a yellow solid.
^{1}H-NMR ($CDCl_3$, 400 MHz): δ 3.89 (s, 3H, OCH_3); 4.78 (d, 1H, *J* = 11.75 Hz, CHC=O); 5.29 (d, 1H, *J* = 11.73 Hz, Ph-CH); 7.28 (d, 2H, *J* = 8.45 Hz, CH-3, CH-5 Ph); 7.53 (d, 2H, *J* = 8.58 Hz, CH-2, CH-6 Ph).

***methyl 2,3-dibromo-3-(5-methylthiophen-2-yl)propanoate* 24.3a (path. B)**
TLC CE : EtOAc 9:1, Rf: 0.50
Silica gel column chromatography with CE : EtOAc 7:3 elution.
Yield: 0.178 g (25%) of a yellow solid.
^{1}H -NMR ($CDCl_3$, 400 MHz): δ 2.38 (s, 3H, CH_3-Thio); 3.88 (s, 3H, OCH_3); 4.72 (d, 1H, *J* = 11.49 Hz, CHC=O); 5.51 (d, 1H, *J* = 11.49 Hz, Thio-CH); 6.99 (d, 1H, *J* = 1.90 Hz, CH-4 Thio); 7.26 (d, 1H, *J* = 1.90 Hz, CH-3 Thio).
A byproduct (0.448 g) was also isolated and structurally characterized by NMR analysis:
***methyl 2-bromo-3-(5-methylthiophen-2-yl)acrylate* 24.3b (path. B)**
^{1}H-NMR ($CDCl_3$, 400 MHz): δ 2.54 (s, 3H, CH_3-Thio); 3.88 (s, 3H, OCH_3); 6.83 (d, 1H, *J* = 3.63 Hz, CH-4 Thio); 7.36 (d, 1H, *J* = 3.63 Hz, CH-3 Thio); 8.36 (s, 1H, Thio-CH).

General procedure for the synthesis of *methyl* or *ethyl 2-bromo-3-arylacrylate derivatives*
A solution of the appropriate aldehyde and methyl or ethyl 2-bromoacetate (1.1 eq.) in 10 mL of DCM was subjected to anhydrous conditions, then $TiCl_4$ (1.2 eq.) was added drop by drop. After 30 minutes also triethylamine (TEA, 2 eq.) was added drop by drop. The reaction mixture was stirred for 2 hours at room temperature. Then it was diluted with DCM and washed with HCl 1N. The organic phase was washed with brine, dried over anhydrous Na_2SO_4, filtered and the solvent was concentrated under *vacuum*.

***methyl (E) and (Z)-3-(benzo[d][1,3]dioxol-5-yl)-2-bromoacrylate* 25.3 (path. C)**
TLC CE : EtOAc 8:2, Rf: 0.57
Silica gel column chromatography with CE : EtOAc 8:2 elution.
Yield: 0.450 g (47%) of a pale yellow solid.
^{1}H-NMR ($CDCl_3$, 400 MHz): δ 3.88 (s, 3H, OCH_3); 6.03 (s, 2H, CH_2); 6.84 (d, 1H, *J* = 8.17 Hz, CH-7 Ph); 7.29 (dd, 1H, *J* = 1.78, 8.17 Hz, CH-6 Ph); 7.64 (d, 1H, *J* = 1.65 Hz, CH-4 Ph); 8.13 (s, 1H, CH=C-Br).

***methyl (E) and (Z)-2-bromo-3-(furan-2-yl)acrylate* 26.3 (path. C)**
TLC CE : EtOAc 8:2, Rf: 0.66
Silica gel column chromatography with CE : EtOAc 8:2 elution.
Yield: 0.835 g (69%) of an orange solid.
^{1}H-NMR ($(CD_3)_2CO$, 400 MHz): δ 3.92 (s, 3H, OCH_3); 6.62 (dd, 1H, *J* = 1.74, 3.58 Hz, CH-4 Fur); 7.48 (d, 1H, *J* = 3.58 Hz, CH-3 Fur); 7.65 (d, 1H, *J* = 1.71 Hz, CH-5 Fur); 8.20 (s, 1H, Fur-CH).

***ethyl (E) and (Z)-2-bromo-3-(4-((3,4-dichlorobenzyl)oxy)phenyl)acrylate* 27.3 (path. C)**
TLC CE : EtOAc 8:2, Rf: 0.67
Silica gel column chromatography with CE : EtOAc 8:2 elution.
Yield: 0.631 g (63%) of a yellow solid that was directly used in the next reaction.

Synthesis of *methyl (E) and (Z)-2-bromo-3-(5-bromopyridin-2-yl)acrylate* 28.3 (path. D)
To a solution of methyl 2-bromo-2-(triphenylphosphoranylidene)acetate (**30**) in 9 mL of DCM was placed at 0°C, then 5-bromopirydin-2-yl aldehyde (0.66 eq.) and TEA (5.28 eq.) were added. The reaction mixture was stirred at room temperature overnight. Then it was concentrated under *vacuum*.

TLC CE : EtOAc 8:2, Rf: 0.35
Silica gel column chromatography with CE : EtOAc 8:2 elution.
Yield: 0.284 g (quant.)
^{1}H-NMR ($CDCl_3$, 400 MHz): 3.92 (s, 3H, OCH_3); 7.90 (dd, 1H, J = 2.32, 8.56 Hz, CH-4 Pyr); 8.05 (d, 1H, J = 8.56 Hz, CH-3 Pyr); 8.26 (s, 1H, Pyr-C<u>H</u>); 8.76 (d, 1H, J = 2.32 Hz, CH-6 Pyr).

Synthesis of *methyl 2-(triphenylphosphoranylidene)acetate* 30.1 (path. D)
To a solution of the 2-methoxy-2-oxoethyl)triphenylphosphonium bromide **30.2** in 20 mL of DCM water was added (20 mL) and dropwise a solution of NaOH 1M (1.1 eq.) until pH 14. At the end the two phases were separated and the aqueous one was extracted with DCM. The organic phase was washed with brine saturated solution, dried over anhydrous Na_2SO_4, filtered and the solvent was concentrated under *vacuum*.
TLC DCM : MeOH 9:1, Rf: 0.62
Yield: 0.780 g (97%) of a yellow solid.
^{1}H-NMR ($CDCl_3$, 400 MHz): δ 3.26 (s, 1H, CH=P); 3.52 (s, 3H, OCH_3); 7.42-7.50 (m, 6H, CH Ph); 7.51-7.59 (m, 3H, CH Ph)); 7.60-7.72 (m, 6H, CH Ph).

Synthesis of *methyl 2-bromo-2-(triphenylphosphoranylidene)acetate* 30 (path. D)
To a solution of the methyl 2-(triphenylphosphoranylidene)acetate **30.1** in 10 mL of DCM, a solution of bromine (1.1 eq.) in DCM (3 mL) was added drop by drop. The reaction mixture was stirred for 2 hours at room temperature. After the solvent was concentrated under *vacuum* to eliminate also the possible excess of Br_2.
TLC DCM : MeOH 9:1, Rf: 0.81
Yield: 1.17 g of a yellow solid that was used without further purification.
^{1}H-NMR ($CDCl_3$, 400 MHz): δ 3.73 (s, 3H, OCH_3); 7.63-7.74 (m, 9H, CH Ph); 7.75-7.86 (m, 3H, CH Ph)); 8.06-8.14 (m, 3H, CH Ph).

First general procedure for the synthesis of *methyl 3-arylpropiolate derivatives* as in Pathway B
1. The appropriate methyl (or ethyl) 2,3-dibromo-3-arylpropanoate derivative was added to a solution of KOH (8 eq.) in 20 mL of ethanol. The reaction mixture was stirred and refluxed for two hours. Then it was concentrated under *vacuum* and water was added. This aqueous phase was treated with Et_2O and after separation it was acidified with HCl 6N up to pH 2 and then extracted with EtOAc. The organic phase was dried over anhydrous Na_2SO_4, filtered and the solvent was concentrated under *vacuum*. All the obtained crudes were directly used in the subsequent esterification reaction.
2. For the esterification K_2CO_3 (1.5 eq.) was added to a solution of the appropriate propiolic acid in 15 mL of acetone and the suspension was stirred for some minutes. After dimethyl sulfate was added. The reaction mixture was stirred and refluxed for one hour. Then it was cooled and plenty of Et_2O was added. The obtained suspension was filtered and the filtrate was concentrated under *vacuum*.

***methyl 3-(4-(benzyloxy)phenyl)propiolate* 17.1**
TLC CE : EtOAc 8:2, Rf: 0.40
Silica gel column chromatography with CE : EtOAc 95:5 elution.
Yield: 0.138 g (18%) of a solid.
^{1}H-NMR ($CDCl_3$, 400 MHz): δ 3.82 (s, 3H, OCH_3); 5.09 (s, 2H, CH_2); 6.95 (d, 2H, J = 8.86 Hz, CH-3, CH-5 Ph); 7.39-7.4 (m, 5H, CH Bn); 7.53 (d, 2H, J = 8.85 Hz, CH-2, CH-6 Ph).

***methyl 3-(4-nitrophenyl)propiolate* 18.1**
TLC CE : EtOAc 8:2, Rf: 0.45
Silica gel column chromatography with CE : EtOAc 9:1 elution.

Yield: 0.135 g (32%) of pale yellow solid.
^{1}H-NMR ($CDCl_3$, 400 MHz): δ 3.87 (s, 3H, OCH_3); 7.74 (d, 2H, *J* = 8.81 Hz, CH-2, CH-6 Ph); 8.25 (d, 2H, *J* = 8.81 Hz, CH-3, CH-5 Ph).

***methyl 3-(naphthalen-2-yl)propiolate* 19.1**
This intermediate was not purified and isolated but used directly in the last reaction step.

***methyl 3-(naphthalen-1-yl)propiolate* 20.1**
This intermediate was not purified and isolated but used directly in the last reaction step.

***methyl 3-(furan-2-yl)propionate* 21.1**
TLC CE : EtOAc 8:2, Rf: 0.58
Silica gel column chromatography with CE : EtOAc 9:1 elution.
Yield: 0.023 g (3%) of a brown solid.
^{1}H-NMR ($CDCl_3$, 400 MHz): δ 3.83 (s, 3H, OCH_3); 6.39 (d, 1H, *J* = 3.56 Hz, CH-4 Fur); 6.87 (d, 1H, *J* = 3.56 Hz, CH-3 Fur).

***methyl 3-(3-bromophenyl)proprionate* 22.1**
TLC DCM : MeOH 9:1, Rf: 0.50
Silica gel column chromatography with CE : EtOAc 95:5 elution.
Yield: 0.157 g (33%) of a pale yellow solid.
^{1}H-NMR ($CDCl_3$, 400 MHz): δ 3.85 (s, 3H, OCH_3); 7.26 (t, 1H, *J* = 7.95 Hz, CH-5 Ph); 7.51 (dt, 1H, *J* = 1.31, 7.98 Hz, CH-6 Ph); 7.59 (ddd, 1H, *J* = 1.04, 1.66, 8.09 Hz, CH-4 Ph); 7.73 (t, 1H, *J* = 1.68 Hz, CH-2 Ph).

***methyl 3-(4-bromophenyl)propiolate* 23.2**
The reaction mixture was stirred and refluxed for 4 hours. Then it was concentrated under *vacuum* and water was added. The pH was adjusted to 4-5 value using HCl 1N solution. The obtained solid was filtered off and washed with water. The acid **23.3** was directly used in the subsequent esterification reaction as described at point 2 of the general procedure reported above.
TLC CE : EtOAc 9:1, Rf: 0.62
Automated flash chromatography (*Isolera-Biotage* system) with CE : EtOAc (2 → 10% EtOAc) elution.
Yield: 0.156 g (33%) of a white solid.
^{1}H-NMR ($CDCl_3$, 400 MHz): δ 3.83 (s, 3H, OCH_3); 7.44 (d, 2H, *J* = 8.44 Hz, CH-2, CH-6 Ph); 7.53 (d, 2H, *J* = 8.53 Hz, CH-3, CH-5 Ph).

Second general procedure for the synthesis of *methyl 3-arylpropiolate derivatives* as in Pathway C
1. To a solution of the methyl 2-bromo-3-arylacrylate derivative in 5 mL of THF, sodium amide (1 eq.) and potassium *tert*-butoxide (1 eq.) were added. At the end, the mixture was concentrated under *vacuum* and water was added. This aqueous phase was treated with Et_2O and after separation it was acidified with HCl 6N up to pH 2 and then extracted with EtOAc. The organic phase was dried over anhydrous Na_2SO_4, filtered and the solvent was concentrated under *vacuum*. All the crudes were directly used in the subsequent esterification reaction.
2. For the esterification K_2CO_3 (1.5 eq.) was added to a solution of the appropriate propiolic acid in 15 mL of acetone and the suspension was stirred for some minutes. After dimethyl sulfate was added. The reaction mixture was stirred and refluxed for one hour. Then it was cooled and plenty of Et_2O was added. The obtained suspension was filtered and the filtrate was concentrated under *vacuum*.

***methyl 3-(5-methylthiophen-2-yl)propiolate* 24.1**
Yield: 0.075 g (60%) of a solid.
^{1}H-NMR ($CDCl_3$, 400 MHz): δ 2.38 (s, 3H, Thio-CH_3); 3.70 (s, 3H, OCH_3); 6.70 (d, 1H, *J* = 3.69 Hz, CH-4 Thio); 7.10 (d, 1H, *J* = 3.70 Hz, CH-3 Thio).

***methyl 3-(benzo[d][1,3]dioxol-5-yl)propiolate* 25.1**
TLC CE : EtOAc 8:2, Rf: 0.63
Yield: 0.170 g (quant.) of a yellow solid that was used without further purification.
^{1}H-NMR ($CDCl_3$, 400 MHz): δ 3.68 (s, 3H, OCH_3); 6.07 (s, 2H, CH_2); 6.85 (d, 1H, *J* = 7.70 Hz, CH-7 Ph); 7.00 (d, 1H, *J* = 7.70 Hz, CH-6 Ph); 7.41 (s, 1H, CH-4 Ph).

***methyl 3-(furan-2-yl)propiolate* 26.1**
TLC CE : EtOAc 8:2, Rf: 0.62
Yield: 0.476 g (98%) of an orange oil that was used without further purification.
^{1}H-NMR ($CDCl_3$, 400 MHz): δ 3.23 (s, 1H, OCH_3); 6.47 (dd, 1H, *J* = 1.84, 3.50 Hz, CH-4 Fur); 6.93 (d, 1H, *J* = 3.50 Hz, CH-3 Fur); 7.51 (d, 1H, J = 1.82 Hz, CH-5 Fur).

***methyl 3-(4-((3,4-dichlorobenzyl)oxy)phenyl)propiolate* 27.1**
TLC CE : EtOAc 8:2, Rf: 0.63
Yield: 0.450 g (97%) of a yellow solid that was used without further purification.
^{1}H-NMR ($CDCl_3$, 400 MHz): δ 3.82 (s, 3H, OCH_3); 5.03 (s, 2H, Ph-C$\underline{H_2}$); 6.93 (d, 2H, J = 8.76 Hz, CH-3, CH-5 Ph); 7.24 (dd, 1H, J = 1.78, 8.27 Hz, CH-6 Bn); 7.46 (d, 1H, J = 8.27 Hz, CH-5 Bn); 7.52 (d, 1H, *J* = 1.78 Hz, CH-2 Bn); 7.54 (d, 2H, J = 8.76 Hz, CH-2, CH-6 Ph).

***methyl 3-(5-bromopyridin-2-yl)propiolate* 28.1 (path. D)**
Yield: 0.150 g (71%) of a yellow solid that was used without further purification.
^{1}H-NMR ($CDCl_3$, 400 MHz): δ 3.68 (s, 3H, OCH_3); 7.29 (d, 1H, *J* = 8.30 Hz, CH-3 Pyr); 7.95 (d, 1H, *J* = 8.30 Hz, CH-4 Pyr); 8.49 (s, 1H, CH-6 Pyr).

First general procedure for the synthesis of *5-phenylisoxazol-3-ol derivatives* as in Pathway A
NaOH (3.5 eq.) was solubilized in EtOH (10 mL), then $NH_2OH{\cdot}HCl$ (1.5 eq) and dropwise the appropriate dibromo-derivative were added. The reaction mixture was stirred and refluxed for three hours. Then the mixture was concentrated and neutralized with HCl 1N until slight acidity was reached. The suspension was filtered (**14**) or extracted with Et_2O (**15** and **16**). The organic phase was washed with brine saturated solution, dried over anhydrous Na_2SO_4, filtered and the solvent was concentrated under *vacuum*.

***5-(3-trifluoromethylphenyl)isoxazol-3-ol* 14**
TLC DCM : MeOH 9:1, Rf: 0.56
Silica gel column chromatography with CE : EtOAc 6:4 elution.
Yield: 0.150 g (33%) of a white solid; m.p. = 188-190°C
^{1}H-NMR ($(CD_3)_2CO$, 400 MHz): δ 6.74 (s, 1H, CH-4 Isox); 7.82 (t, 1H, *J* = 7.90 Hz, CH-5 Ph); 7.88 (d, 1H, *J* = 7.90 Hz, CH-4 Ph); 8.15 (d, 2H, *J* = 7.90 Hz, CH-2, CH-6 Ph); 10.25 (s, 1H, OH).
^{13}C-NMR ($(CD_3)_2CO$, 400 MHz): 93.3 (C-4 Isox); 122.8 (C-1 Ph); 123.0 (C-2 Ph); 127.4 (C-3 Ph); 127.7 (C-4 Ph); 129.7 (CF_3); 129.8 (C-6 Ph); 131.3 (C-5 Ph); 169.3 (C-5 Isox); 172.3 (C-3 Isox).
HRMS m/z $[M+H]^+$ calcd. for $C_{10}H_7F_3NO_2^+$: 230.0423; found: 230.0421.

***5-(2-clorophenyl)isoxazol-3-ol* 15**
TLC DCM : MeOH 9:1, Rf: 0.76
Silica gel column chromatography with DCM : MeOH 98:2 elution.

The product was still not pure, so DCM was added and the resulting precipitate was left to settle overnight. The suspension was filtered and the solid was collected.
Yield: 0.035 g (7.7%) of a white solid; m.p. = 164-165°C
^{1}H-NMR (($(CD_3)_2CO$, 400 MHz): δ 6.66 (s, 1H, CH-4 Isox); 7.55-7.57 (m, 2H, CH-4, CH-5 Ph); 7.65-7.67 (m, 1H, CH-3 Ph); 7.93-7.95 (m, 1H, CH-6 Ph); 10.29 (s, 1H, OH).
^{13}C-NMR ($(CD_3)_2CO$, 400 MHz): δ 96.8 (C-4 Isox); 122.2 (C-5 Ph); 128.5 (C-6 Ph); 130.1 (C-3 Ph); 131.7 (C-4 Ph); 132.2 (C-2 Ph); 140.5 (C-1 Ph); 167 (C-5 Isox); 171.5 (C-3 Isox).
HRMS m/z $[M+H]^+$ calcd. for $C_9H_7ClNO_2^+$: 196.0160; found: 196.0159.

***5-(4-methoxyphenyl)isoxazol-3-ol* 16**
TLC DCM : MeOH 9:1, Rf: 0.46
Silica gel column chromatography with DCM : MeOH 95:5 elution.
Since the product was still not pure, it was crystallized from Et_2O.
Yield: 0.019 g (3%) of a pale-yellow solid; m.p. = 194°C
^{1}H-NMR ($(CD_3)_2CO$, 400 MHz): δ 3.91 (s, 1H, OCH_3); 6.35 (s, 1H, CH-4 Isox); 7.10 (dd, 2H, *J* = 1.93, 6.86 Hz, CH-3, CH-5 Ph); 7.78 (dd, 2H, *J* = 1.79, 6.86 Hz, CH-2, CH-6 Ph); 9.97 (s, 1H, OH).
^{13}C-NMR ($(CD_3)_2CO$, 400 MHz): δ 55.8 (CH_3); 90.6 (C-4 Isox); 119.3 (C-3, C-5 Ph); 121.7 (C-1 Ph); 128.1 (C-2, C-6 Ph); 162.2 (C-4 Ph); 170.8 (C-5 Isox); 171.7 (C-3 Isox).
HRMS m/z $[M+H]^+$ calcd. for $C_{10}H_{10}NO_3^+$: 192.0655; found: 192.0654.

Second general procedure for the synthesis of *5-arylisoxazol-3-ol derivatives* as in Pathways B, C and D
The appropriate ester was solubilized in 0.2-10 mL of MeOH. This solution was added to a NaOH solution (8 eq.) in aq. NH_2OH 50% (1 mL NH_2OH x 0.35 mmol ester). The reaction mixture was stirred at room temperature or heated until 60°C. The reaction time varied from 1 hour to overnight. At the end, the mixture was quenched with acetic acid (8 eq) and concentrated under *vacuum*. The crude was washed with water and extracted with EtOAc. The organic phase was then washed with brine saturated solution, dried over anhydrous Na_2SO_4, filtered and the solvent was concentrated under *vacuum*.

***5-(4-(benzyloxy)phenyl)isoxazol-3-ol* 17**
Reaction at room temperature.
TLC DCM : MeOH 9:1, Rf: 0.63
The title compound was obtained by crystallization from Et_2O.
Yield: 0.055 g (40%) of a solid; m.p. = 200-201°C
^{1}H-NMR ($(CD_3)_2CO$, 400 MHz): δ 5.22 (s, 2H, CH_2); 6.34 (s, 1H, CH-4 Isox); 7.15 (dd, 2H, *J* = 2.02, 6.88 Hz, CH-3, CH-5 Ph); 7.34-7.44 (m, 3H, CH-3, CH-4, CH-5 Bn); 7.51 (d, 2H, *J* = 7.15 Hz, CH-2, CH-6 Bn); 7.76 (dd, 2H, *J* = 1.79, 6.88 Hz, CH-2, CH-6 Ph).
^{13}C-NMR ($(CD_3)_2CO$, 400 MHz): δ 70.7 (CH_2); 90.57 (C-4 Isox); 116.2 (C-3, C-5 Ph); 121.8 (C-1 Ph); 127.9 (C-2, C-6 Ph); 128.5 (C-2, C-6 Bn); 128.8 (C-4 Bn); 129.3 (C-3, C-5 Bn); 137.9 (C-1 Bn); 161.2 (C-4 Ph); 170.1 (C-3, C-5 Isox).
HRMS m/z $[M+H]^+$ calcd. for $C_{16}H_{14}NO_3^+$: 268.0968; found: 268.0965.

***5-(4-nitrophenyl)isoxazol-3-ol* 18**
Reaction at room temperature.
TLC 9:1 DCM : MeOH, Rf: 0.53
Yield: 0.095 g (69%) of an orange solid; m.p. = 155-157°C
^{1}H-NMR ($(CD_3)_2CO$, 400 MHz): δ 6.49 (s, 1H, CH-4 Isox); 7.51-7.56 (m, 2H, CH-3, CH-5 Ph); 7.84 (dd, 2H, *J* = 1.71, 8.07 Hz, CH-2, CH-6 Ph); 10.03 (s, 1H, OH).
^{13}C-NMR ($(CD_3)_2CO$, 400 MHz): δ 92 (C-4 Isox); 126 (C-2, C-6 Ph); 128.9 (C-1 Ph); 129.9 (C-3, C-5 Ph); 131.1 (C-4 Ph); 170.7 (C-5 Isox); 171.8 (C-3 Isox).

HRMS m/z $[M+H]^+$ calcd. for $C_9H_7N_2O_4^+$: 207.0400; found: 207.0401.

***5-(naphthalen-2-yl)isoxazol-3-ol* 19**
Reaction at room temperature.
TLC DCM : MeOH 9:1, Rf: 0.36
Silica gel column chromatography with DCM : MeOH 95:5 elution.
Yield: 0.055 g (22%) of a light brown solid; m.p. = 207-208°C
^{1}H-NMR (($CD_3)_2$CO, 400 MHz): δ 6.63 (s, 1H, CH-4 Isox); 7.63-7.66 (m, 2H, CH-6, CH-7 Naph); 7.94 (dd, 1H, J = 1.76, 8.72 Hz, CH-3 Naph); 8.0 (dd, 1H, J = 3.52, 6.19 Hz, CH-4 Naph); 8.08 (d, 2H, J = 8.10 Hz, CH-5, CH-8 Naph); 8.43 (s, 1H, CH-1 Naph).
^{13}C-NMR (($CD_3)_2$CO, 400 MHz): δ 92.3 (C-4 Isox); 123.5 (C-3 Naph); 125.7 (C-1 Naph); 126.2 (C-4a Naph); 128.2 (C-6, C-7 Naph); 128.7 (C-4 Naph); 129.5 (C-5, C-8 Naph); 134.1 (C-8a Naph); 134.9 (C-2 Naph); 170.1 (C-5 Isox); 171.9 (C-3 Isox).
HRMS m/z $[M+H]^+$ calcd. for $C_{13}H_{10}NO_2^+$: 212.0706; found: 212.0704.

***5-(naphthalen-1-yl)isoxazol-3-ol* 20**
Reaction at room temperature.
TLC DCM : MeOH 9:1, Rf: 0.59
Silica gel column chromatography with DCM : MeOH 95:5 elution.
Yield: 0.080 g (29%) of a pale-yellow solid; m.p. = 180-181°C
^{1}H-NMR (($CD_3)_2$CO, 400 MHz): δ 6.51 (s, 1H, CH-4 isox); 7.68 (m, 3H, CH-3, CH-6, CH-7 Naph); 7.89 (dd, 1H, J = 1.17, 7.22 Hz, CH-4 Naph); 8.07 (dd, 1H, J = 2.12, 7.74 Hz, CH-5 Naph); 8.13 (d, 1H, J = 8.34 Hz, CH-2 Naph); 8.38 (dd, 1H, J = 0.85, 8.05 Hz, CH-8 Naph).
^{13}C-NMR (($CD_3)_2$CO, 400 MHz): δ 96 (C-4 Isox); 125.8 (C-2 Naph); 126.2 (C-3 Naph); 126.6 (C-8a Naph); 127.4 (C-6, C-7 Naph); 128.3 (C-4, C-5, C-8 Naph); 129.6 (C-4a Naph); 131 (C-1 Naph); 170.9 (C-5 Isox); 171.5 (C-3 Isox).
HRMS m/z $[M+H]^+$ calcd. for $C_{13}H_{10}NO_2^+$: 212.0706; found: 212.0705.

***5-(5-bromofuran-2-yl)isoxazol-3-ol* 21**
Reaction at room temperature.
TLC DCM : MeOH 9:1, Rf: 0.63
Yield: 0.020 g (97%) of a pale orange solid; m.p. = 144-145°C
^{1}H-NMR (($CD_3)_2$CO, 400 MHz): δ 6.33 (s, 1H, CH-4 Isox); 6.75 (d, 1H, J = 3.54 Hz, CH-4 Fur); 7.03 (d, 1H, J = 3.54 Hz, CH-3 Fur) 10.21 (s, 1H, OH).
^{13}C-NMR (($CD_3)_2$CO, 400 MHz): δ 95.1 (C-4 Isox); 105.1 (C-4 Fur); 109.4 (C-5 Fur); 119.5 (C-3 Fur); 156.7 (C-2 Fur); 167.5 (C-5 Isox); 171.5 (C-3 Isox).
HRMS m/z $[M+H]^+$ calcd. for $C_7H_5BrNO_3^+$: 229.9447; found: 229.9447.

***5-(3-bromophenyl)isoxazol-3-ol* 22**
Reaction from r.t. to 60°C.
TLC DCM : MeOH 9:1, Rf: 0.49
Yield: 0.069 g (43%) of a pale pink solid; m.p. = 195°C
^{1}H-NMR (($CD_3)_2$CO, 400 MHz): δ 6.63 (s, 1H, CH-4 Isox); 7.52 (t, 1H, J = 7.78 Hz, CH-5 Ph); 7.70 (ddd, 1H, J = 1.01, 2.04, 8.06 Hz, CH-6 Ph); 7.86 (dt, 1H, J = 1.08, 7.84 Hz, CH-4 Ph); 8.02 (t, 1H, J = 1.75 Hz, CH-2 Ph); 10.1 (s, 1H, OH).
^{13}C-NMR (($CD_3)_2$CO, 400 MHz): δ 93.2 (C-4 Isox); 123.4 (C-3 Ph); 125 (C-6 Ph); 128.9 (C-4 Ph); 130.9 (C-5 Ph); 131.9 (C-1 Ph); 133.8 (C-2 Ph); 168.9 (C-5 Isox); 171.9 (C-3 Isox).
HRMS m/z $[M+H]^+$ calcd. for $C_9H_7BrNO_2^+$: 239.9655; found: 239.9654.

***5-(5-methylthiophen-2-yl)isoxazol-3-ol* 24**
Reaction from r.t. to 60°C.

TLC DCM : MeOH 9:1, Rf: 0.52
Yield: 0.045 g (74%) of a yellow solid; m.p. = 156-157°C
^{1}H-NMR ((CD_3)$_2$CO, 400 MHz): δ 2.57 (s, 3H, CH_3); 6.23 (s, 1H, CH-4 Isox); 6.92 (m, 1H, CH-4 Thio); 7.41 (d, 1H, *J* = 3.63 Hz, CH-3 Thio); 10.01 (s, 1H, OH).
^{13}C-NMR ((CD_3)$_2$CO, 400 MHz): δ 15.15 (CH_3); 90.6 (C-4 Isox); 127.5 (C-4 Thio); 127.9 (C-3 Thio); 128.2 (C-2 Thio); 143.95 (C-5 Thio); 166.1 (C-5 Isox); 171.6 (C-3 Isox).
HRMS m/z $[M+H]^+$ calcd. for $C_8H_8NO_2S^+$: 182.0270; found: 182.0269.

***5-(benzo[d][1,3]dioxol-5-yl)isoxazol-3-ol* 25**
Reaction from r.t. to 60°C.
TLC DCM : MeOH 9:1, Rf: 0.60
Yield: 0.138 g (81%) of a white solid; m.p. = 212-213°C
^{1}H-NMR ((CD_3)$_2$CO, 400 MHz): δ 6.14 (s, 2H, CH_2); 6.38 (s, 1H, CH-4 Isox); 7.01 (d, 1H, *J* = 8.19 Hz, CH-7 Ph); 7.31 (dd, 1H, *J* = 1.71 Hz, CH-4 Ph); 7.39 (dd, 1H, *J* = 1.71, 8.19 Hz, CH-6 Ph); 9.97 (s, 1H, OH).
^{13}C-NMR ((CD_3)$_2$CO, 400 MHz): δ 90.9 (C-4 Isox); 102.8 (CH_2); 106.4 (C-4 Pip); 109.5 (C-7 Pip); 120.8 (C-6 Pip); 123 (C-5 Pip); 149.3 (C-7a Pip); 150.2 (C-3a Pip); 170.6 (C-5 Isox); 171.7 (C-3 Isox).
HRMS m/z $[M+H]^+$ calcd. for $C_{10}H_8NO_4^+$: 206.0448; found: 206.0447.

***5-(furan-2-yl)isoxazol-3-ol* 26**
Reaction from r.t. to 60°C.
TLC DCM : MeOH 9:1, Rf: 0.59
Yield: 0.413 g (86%) of a light red solid; m.p. = 181-182°C
^{1}H-NMR ((CD_3)$_2$CO, 400 MHz): δ 6.27 (s, 1H, CH-4 Isox); 6.69 (dd, 1H, *J* = 1.84, 3.46 Hz, CH-4 Fur); 7.01 (d, 1H, *J* = 3.46 Hz, CH-3 Fur); 7.80 (dd, *J* = 0.56, 1.79 Hz, CH-5 Fur); 10.1 (s, 1H, OH).
^{13}C-NMR ((CD_3)$_2$CO, 400 MHz): δ 91.26 (C-4 Isox); 110.9 (C-3 Fur); 112.8 (C-4 Fur); 144 (C-2 Fur); 145.5 (C-5 Fur); 162 (C-5 Isox); 171 (C-3 Isox).
HRMS m/z $[M+H]^+$ calcd. for $C_7H_6NO_3^+$: 152.0342; found: 152.0341.

***5-(4-((3,4-dichlorobenzyl)oxy)phenyl)isoxazol-3-ol* 27**
Reaction from r.t. to 60°C.
TLC DCM : MeOH 9:1, Rf: 0.54
Yield: 0.360 g (80%) of a white solid; m.p. = 202-203°C
^{1}H-NMR ((CD_3)$_2$CO, 400 MHz): δ 5.29 (s, 2H, CH_2); 6.36 (s, 1H, CH-4 Isox); 7.20 (d, 2H, *J* = 8.87 Hz, CH-3, CH-5 Ph); 7.54 (dd, 1H, *J* = 1.90, 8.29 Hz, CH-6 Bn); 7.67 (d, 1H, *J* = 8.29 Hz, CH-5 Bn); 7.78 (d, 1H, *J* = 1.90 Hz, CH-2 Bn); 7.81 (d, 2H, *J* = 8.87 Hz, CH-2, CH-6 Ph); 9.98 (s, 1H, OH).
^{13}C-NMR ((CD_3)$_2$CO, 400 MHz): δ 69.1 (CH_2); 90.6 (C-4 Isox); 116.2 (C-3, C-5 Ph); 122.1 (C-1 Ph); 127.9 (C-2, C-6 Ph); 128.4 (C-6 Bn); 130.4 (C-2 Bn); 131.6 (C-5 Bn); 132.1 (C-3 Bn); 132.8 (C-4 Bn); 139.1 (C-1 Bn); 160.8 (C-4 Ph); 170.7 (C-5 Isox); 171.1 (C-3 Isox).
HRMS m/z $[M+H]^+$ calcd. for $C_{16}H_{12}Cl_2NO_3^+$: 336.0189; found: 336.0190.

***5-(5-bromopyridin-2-yl)isoxazol-3-ol* 28**
Reaction from r.t. to 60°C.
Silica gel column chromatography with DCM : MeOH 9:1 elution.
Yield: 0.025 g (16%) of a white solid; m.p. = 183-184°C
^{1}H-NMR ((CD_3)$_2$CO, 400 MHz): δ 6.65 (s, 1H, CH-4 Isox); 7.88 (d, 1H, *J* = 8.39 Hz, CH-3 Pyr); 8.23 (dd, 1H, *J* = 2.38, 8.40 Hz, CH-4 Pyr); 8.82 (d, 1H, *J* = 2.38 Hz, CH-6 Pyr).

^{13}C-NMR (($(CD_3)_2CO$, 400 MHz): δ 94.7 (C-4 Isox); 122.1 (C-5 Pyr); 122.4 (C-3 Pyr); 140.8 (C-4 Pyr); 146.2 (C-2 Pyr); 152 (C-6 Pyr); 169.5 (C-5 Isox); 171.9 (C-3 Isox).
HRMS m/z $[M+H]^+$ calcd. for $C_8H_6BrN_2O_2^+$: 240.9607; found: 240.9607.

Synthesis of *5-(4-bromophenyl)isoxazol-3-ol* 23.1 (path. B)
NaOH (3.5 eq.) was solubilized in 10 mL of EtOH, then $NH_2OH{\cdot}HCl$ and dropwise the methyl 3-(4-bromophenyl)propiolate **23.2** (1.5 eq) were added. The reaction mixture was stirred and refluxed for 3 hours. Then it was concentrated and neutralized with HCl 1N until slight acidity was reached. The suspension was extracted with EtOAc. The organic phase was washed with brine saturated solution, dried over anhydrous Na_2SO_4, filtered and the solvent was concentrated under *vacuum*.
TLC DCM : MeOH 9:1, Rf: 0.61
Yield: 0.110 g (70%) of a yellow solid.
^{1}H-NMR (DMSO-d_6, 400 MHz): δ 6.61 (s, 1H, CH-4 Isox); 7.71 (d, 2H, J = 8.80 Hz, CH-3, CH-5 Ph); 7.75 (d, 2H, J = 8.87 Hz, CH-2, CH-6 Ph); 11.43 (s, 1H, OH).

Synthesis of *5-(4-(benzylamino)phenyl)isoxazol-3-ol* 23 (path. B)
To the solution of 5-(4-bromophenyl)isoxazol-3-ol in dry toluene benzylamine, K_2CO_3, xPhos and tris(dibenzylideneacetone)dipalladium(0) were added. The mixture was refluxed for 8 hours. Then it was cooled, and the solvent was removed under vacuum. The residue was dissolved in EtOAc and the organic phase was washed with water and brine saturated solution, dried over anhydrous Na_2SO_4, filtered and the solvent was concentrated under vacuum. The desired product was crystallized from Et_2O and it was obtained as a white solid.
TLC DCM : MeOH 9:1, Rf: 0.46
Yield: 0.014 g (9%); m.p. = 204-205°C
^{1}H-NMR (DMSO-d_6, 400 MHz): δ 5.18 (s, 2H, CH_2); 6.35 (s, 1H, CH-4 Isox); 7.35 (m, 3H, CH-3, CH-5 Ph, NH); 7.38-7.43 (m, 3H, CH-3, CH-4, CH-5 Bn); 7.47 (d, 2H, J = 6.87 Hz, CH-2, CH-6 Bn); 7.72 (d, 2H, J = 8.18 Hz, CH-2, CH-6 Ph).
^{13}C-NMR (DMSO-d_6, 400 MHz): δ 69.3 (CH_2); 90.77 (C-4 Isox); 115.26 (C-3, C-5 Ph); 120.54 (C-1 Ph); 126.80 (C-2, C-6 Ph); 127.85 (C-2, C-6 Bn); 127.95 (C-4 Bn); 128.45 (C-3, C-5 Bn); 136.66 (C-1 Bn); 159.6 (C-4 Ph); 171.50 (C-3, C-5 Isox).
HRMS m/z $[M+H]^+$ calcd. for $C_{16}H_{15}N_2O_2^+$: 267.1128; found: 267.1120.

Synthesis of *ethyl 5-phenylisoxazole-3-carboxylate* 29.1 (path. E)
To a solution of ethyl 2,4-dioxo-4-phenylbutanoate in 10 mL of EtOH, $NH_2OH{\cdot}HCl$ (2.5 eq.) was added. The reaction mixture was stirred and refluxed for two hours. Then it was concentrated and the residue treated in an ice bath. The precipitate obtained was recovered by filtration.
TLC CE : EtOAc 1:1, Rf: 0.62
Yield: 0.250 g (quant.) of a pale-yellow solid that was used without further purification.
^{1}H-NMR ($CDCl_3$, 400 MHz): δ 1.44 (t, 3H, J = 7.10 Hz, $C\underline{H}_3CH_2O$); 4.45 (q, 2H, J = 7.10 Hz, $CH_3C\underline{H}_2O$); 6.92 (s, 1H, CH-4 Isox); 7.47-7.49 (m, 3H, CH-3, CH-4, CH-5 Ph); 7.79-7,.81 (m, 2H, CH-2, CH-6 Ph).

Synthesis of *5-phenylisoaxazol-3-carboxylic acid* 29 (path. E)
To a solution of **29.1** in 10 mL of EtOH, a solution of NaOH 0.1 M was added. The reaction mixture was stirred at room temperature for 1 hour. Then it was concentrated and water was added. The aqueous phase was treated with Et_2O and then acidified with HCl 1N until pH 1. A precipitate formed and it was recovered by filtration.
TLC DCM : MeOH 9:1, Rf: 0.26
Yield: 0.152 g (70%) of a white solid; m.p. = 159°C
^{1}H-NMR ($CDCl_3$, 400 MHz): δ 7.28 (s, 1H, CH-4 Isox); 7.61-7.63 (m, 3H, CH-3, CH-4, CH-5 Ph); 7.99-8.02 (m, 2H, CH-2, CH-6 Ph).

^{13}C-NMR ($(CD_3)_2CO$, 400 MHz): δ 95.2 (C-4 Isox); 125.2 (C-2, C-6 Ph); 128.7 (C-4 Ph); 129.2 (C-3, C-5 Ph); 150 (C-3 Isox); 168.3 (C=O); 169.3 (C-5 Isox).
HRMS m/z $[M+H]^+$ calcd. for $C_{10}H_8NO_3^+$: 190.0499; found: 190.0497.

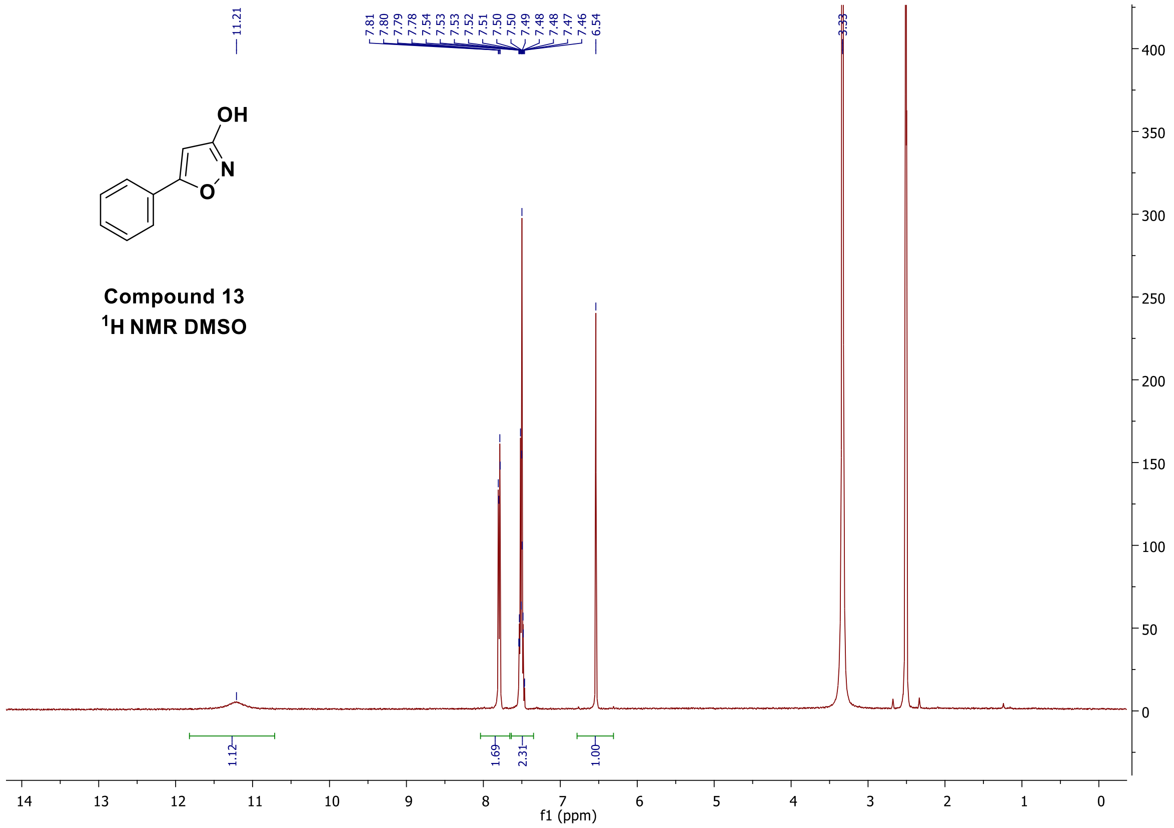

OH
N
O
Compound 13
^{1}H NMR DMSO
11.21
7.81 7.80 7.79 7.78 7.54 7.53 7.53 7.52 7.51 7.50 7.50 7.49 7.48 7.48 7.47 7.46
6.54
3.33
1.12
1.69
2.31
1.00
f1 (ppm)

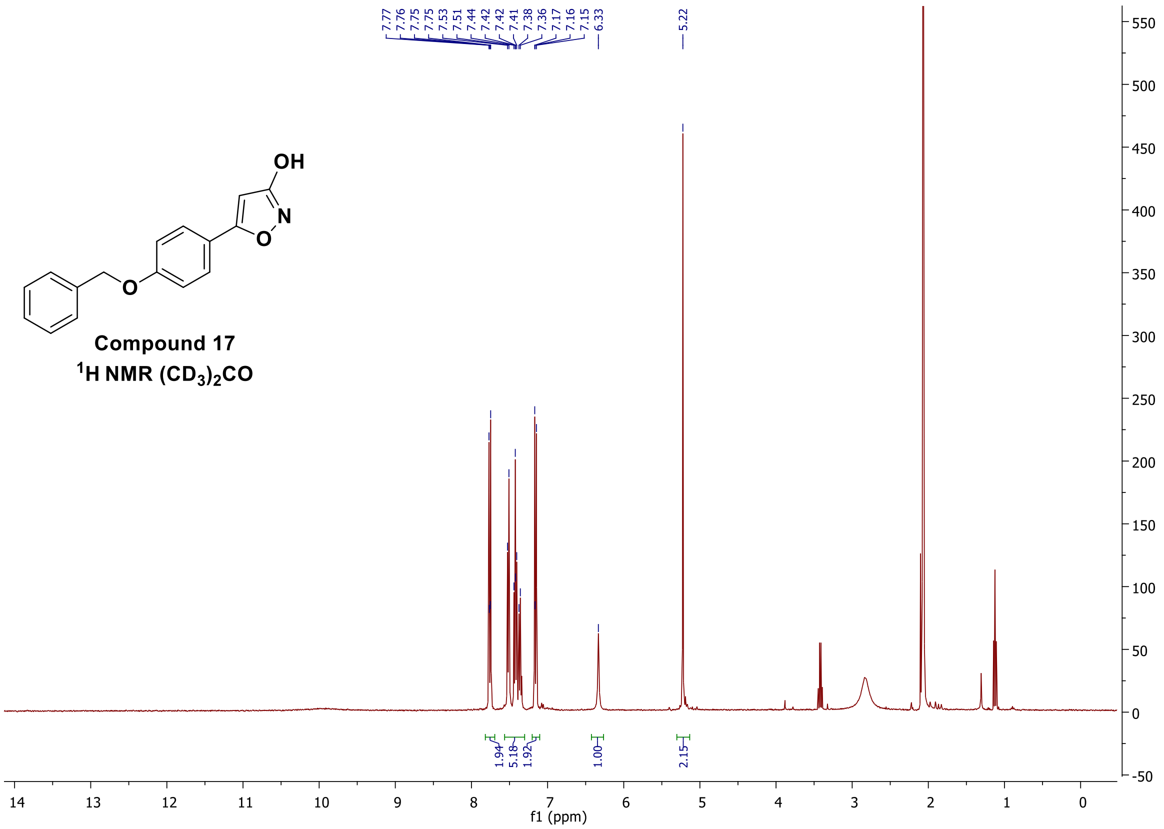

OH
N
O
O
Compound 17
^{1}H NMR $(CD_3)_2CO$
7.77 7.76 7.75 7.75 7.53 7.51 7.44 7.42 7.41 7.38 7.36 7.17 7.15
6.33
5.22
1.94
5.18
1.92
1.00
2.15
f1 (ppm)

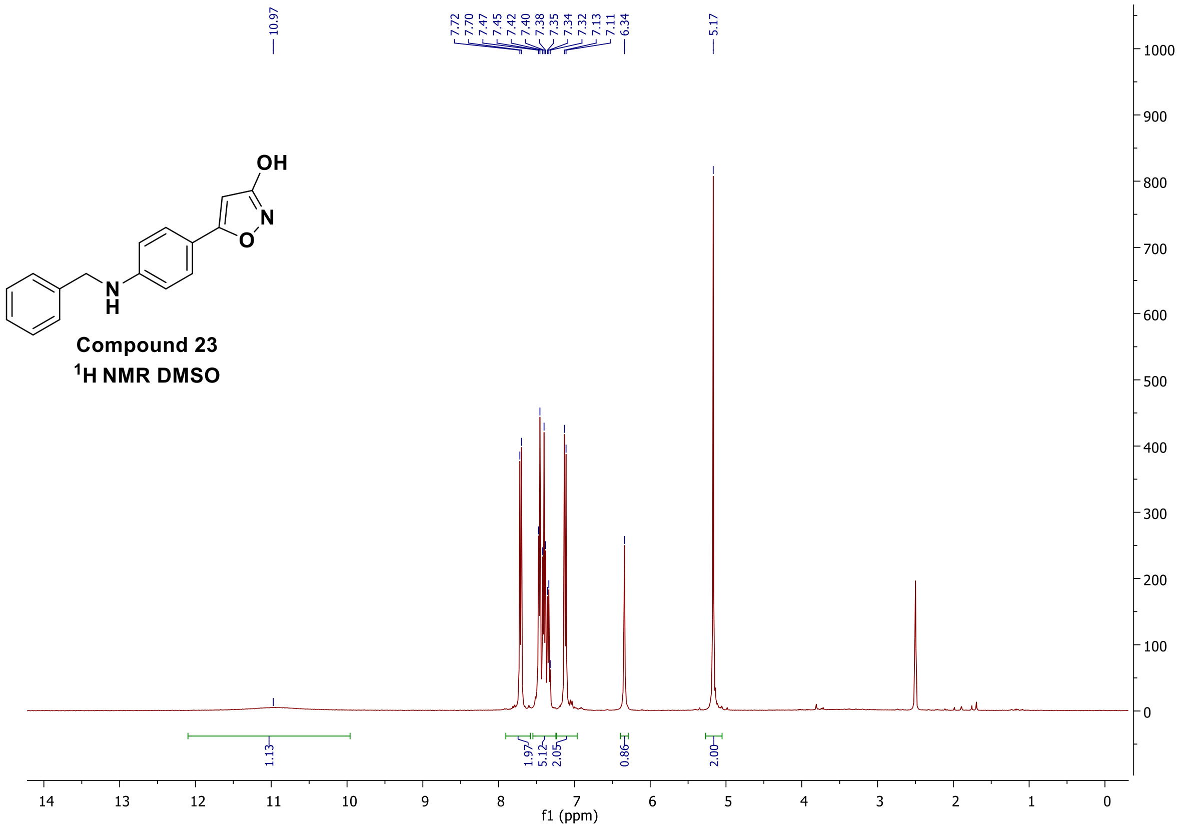
OH
N
O
N
H
Compound 23
1H NMR DMSO
10.97
7.72 7.70 7.47 7.45 7.42 7.40 7.38 7.35 7.34 7.32 7.13 7.11 6.34
5.17
1.13
1.97 5.12 2.05
0.86
2.00
f1 (ppm)

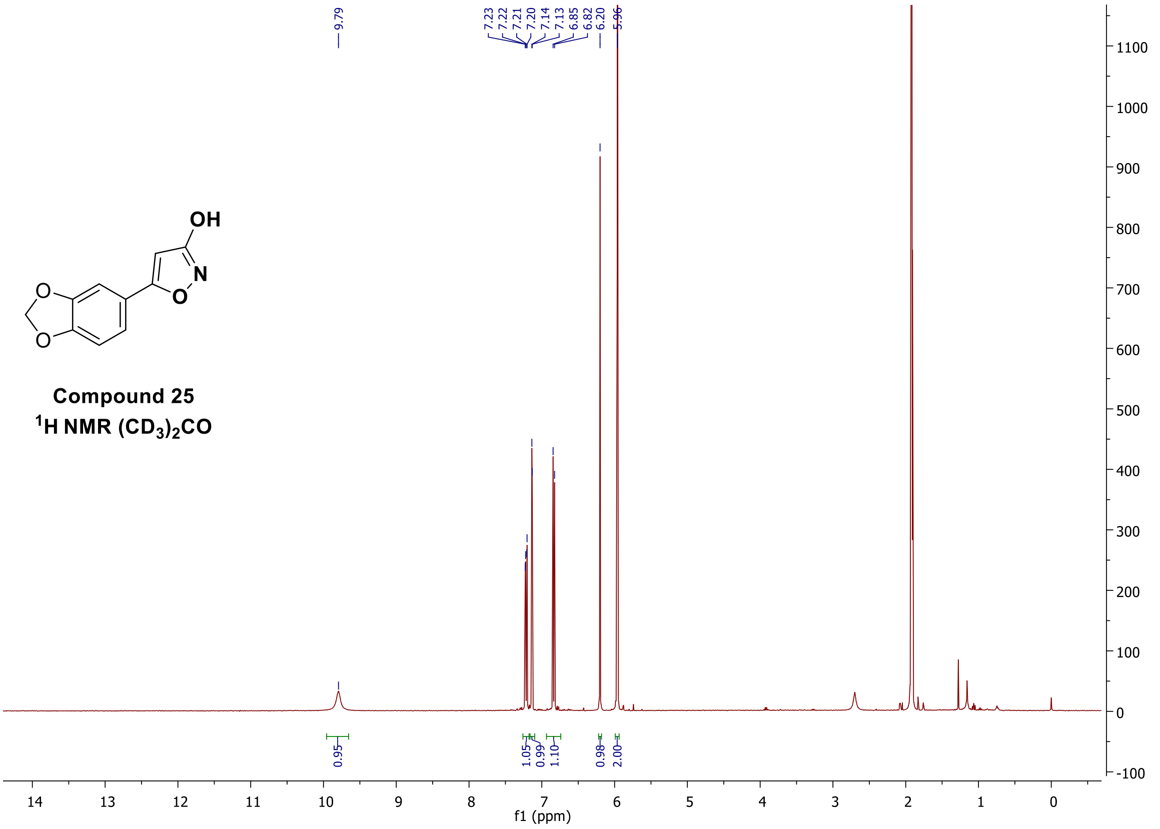
OH
N
O
O
O
Compound 25
1H NMR (CD3)2CO
9.79
7.23 7.22 7.21 7.20 7.14 7.13 6.85 6.82 6.20 5.96
0.95
1.05 0.99 1.10
0.98 2.00
f1 (ppm)

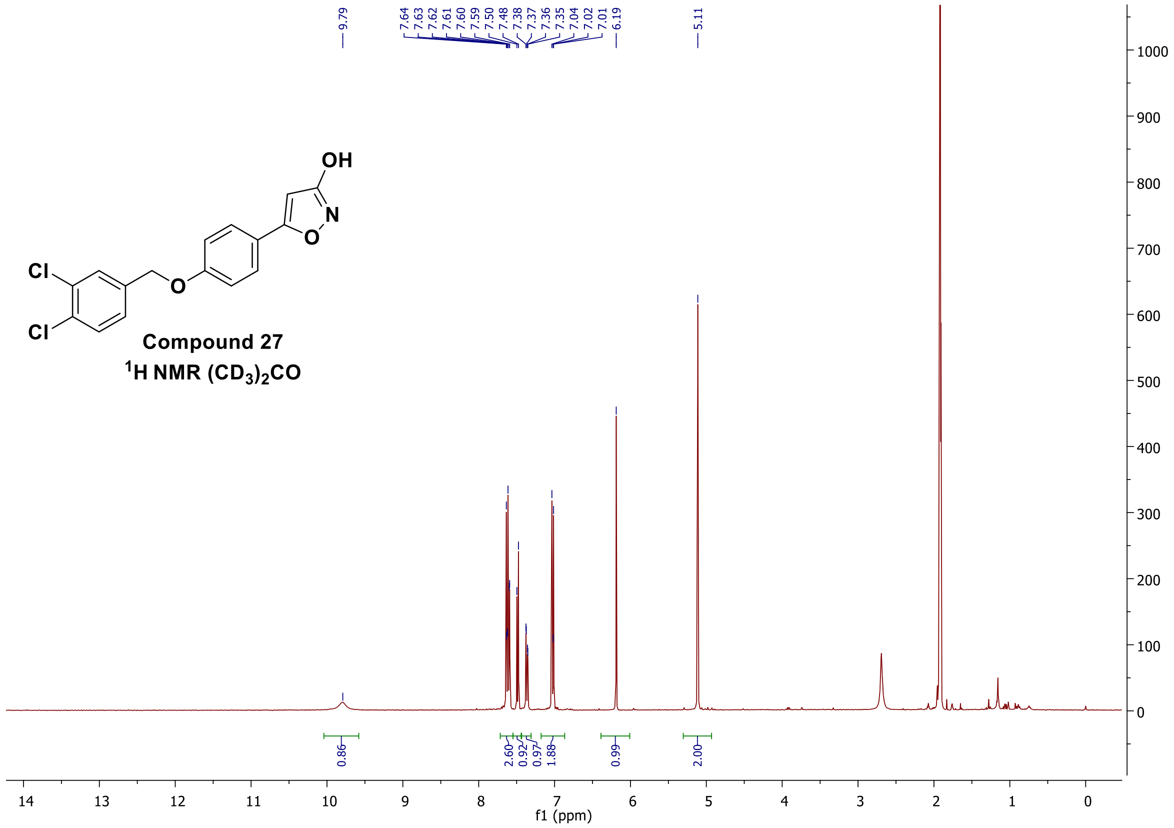
9.79
7.64
7.63
7.62
7.61
7.60
7.59
7.50
7.48
7.38
7.37
7.36
7.35
7.04
7.02
7.01
6.19
5.11
OH
N
O
Cl
Cl
O
Compound 27
1H NMR (CD3)2CO
0.86
2.60
0.92
0.97
1.88
0.99
2.00
f1 (ppm)

1.3 In Vitro HDAC6 Assay

Enzymatic HDAC6 assays were performed with a Fluorogenic Assay Kit (#50076, BPS Bioscience) following the manufacturer's protocol, with slight modifications. Briefly, 25µl reaction mixtures were prepared in low binding 96-wells black plates in HDAC buffer containing 100µg/ml of BSA, 0,25ng/µl of HDAC6 human recombinant enzyme, 50 µM HDAC substrate and 2µl of test inhibitor. Compounds were tested in single dose, with 3-fold serial dilutions starting at 300µM. Trichostatin A (TSA) was used as a control inhibitor. The resulting mixtures were incubated at 37 °C for 45 min, after which 25 µL of 2X HDAC developer were added. Following incubation at RT for 15 min, fluorescence intensity was measured using a microplate reader (GloMax Discover System, Promega) at 365nm excitation and 415-44 nm emission wavelengths.
IC_{50} values were calculated using the GraphPad Prism 6 program based on a log(inhibitor) vs response (variable slope) sigmoidal equation. Curve fits were performed for data sets that displayed less than 55% enzyme activity at the highest compound concentration tested.

1.4 Western blot analysis

Cell Line
The prostate cancer cell line DU145 (ATCC) was grown in RPMI medium (Euroclone, Milan, Italy) with 10% fetal bovine serum (Euroclone), 2 mM L-glutamine (Euroclone), and antibiotics (100 U/mL penicillin, 100 L g/mL streptomycin) (Euroclone). Cells were stimulated with drugs for 24, 48, 72 hours at 50, 10, 5 and 1 µM.

Total Protein Extraction and western blot
After the induction with the test compounds at the indicated times and concentrations, DU145 cells were then harvested, washed with phosphate-buffered saline (PBS) (Euroclone), and lysed for 15 min at 4 °C in lysis extraction buffer with protease and phosphatase inhibitors (50 mM Tris-HCl pH 8.0, 150 mM NaCl, 1% NP40, 10 mM sodium fluoride, 0.1 mM sodium orthovanadate, 40 mg/mL phenylmethylsulfonyl fluoride 58 (PMSF), 20 g/mL aprotinin, 20 mg/mL leupeptin, 2 mg/mL antipain, 10 mM p-nitrophenyl phosphate, 10 mg/mL pepstatin A and 20 nM okadaic acid). Cells were vortexed then centrifuged at 13000 rpm for 30 min at 4 °C. Bradford assay (Biorad, Italy) was used to quantify protein concentration. Cell extracts were 1:1 diluted in the 2× Laemmli sample buffer (0.217 M Tris–HCl with pH 8.0, 52.17% SDS, 17.4% glycerol, 0.026% bromophenol blue, and 8.7% β-mercaptoethanol), and then boiled for 3 min. Equal amounts of protein (35 µg) were run, separated by SDS–PAGE gel, transferred onto nitrocellulose membranes, and incubated with primary antibodies. Cell extracts were separated by SDS-PAGE and western blots were carried out for acetyl-tubulin (Sigma) and anti-GAPDH antibody (Santa Cruz Biotechnology) as the loading control. The immune complexes were first detected with horseradish peroxidase-conjugated species-specific secondary antiserum (Bio-Rad Laboratories, Milan, Italy), and then by an enhanced chemiluminescence reaction (Bio-Rad Laboratories, Milan, Italy). Densitometric analysis of protein expression was performed by using ImageJ image processing package.

Histone Extraction and western blot
After the induction with the test compounds at the indicated times and concentrations, DU145 cells were harvested and washed twice with cold 1× PBS and lysed in Triton extraction buffer (TEB; PBS containing 0.5% Triton X 100 (v/v), 2 mM PMSF, and 0.02% (w/v) NaN_3) for 10 min on ice, with gentle stirring. After centrifugation (2000 rpm at 4 °C for 10 min), the supernatant was removed, and the pellet was washed in half the volume of TEB and centrifuged as before. The pellet was overnight incubated in 0.2 N HCl at 4 °C on a rolling table. The samples were then centrifuged at 2000 rpm for 10 min at 4 °C; the concentration of protein in the supernatant was determined

using a Bradford assay (Bio-Rad, CA, USA). Equal amounts of protein (4 μg) were run, separated by SDS–PAGE gel, transferred onto nitrocellulose membranes, and incubated with primary antibodies. For the detection of histone H3 acetylation, H3K9,14ac (Diagenode) was used. Histone H4 (Abcam) antibodies and Pounceau Red (Sigma) were used to normalize for equal loading. Semi-quantitative analysis was performed using ImageJ software.

1.5 Anti-proliferative assay

Cell viability was determined on DU145 cell line using Thiazolyl Blue Tetrazolium Bromide [3-(4,5-dimethylthiazol-2-yl)-2,5-diphenyltetrazolium bromide] (MTT; Sigma-Aldrich) assay after treatment with new compounds at indicated concentrations. A total of 2×10^4 cells/well were plated in a 96-well plate and treated, in duplicates, with new compounds at different concentrations for 24, 48, and 72 hours. Absorbance was read at a wavelength of 570 nm with a TECAN M-200 reader (Tecan, Männedorf, Switzerland).

References

1. Gaulton A, Hersey A, Nowotka M, Bento AP, Chambers J, Mendez D, Mutowo P, Atkinson F, Bellis LJ, Cibrián-Uhalte E, et al. The ChEMBL Database in 2017. Nucleic Acids Res 2017;45(D1):D945–D954.
2. Schrödinger Release 2020-1: LigPrep; Schrödinger, LLC: New York, NY, 2020.
3. Hawkins PCD, Skillman AG, Warren GL, Ellingson BA, Stahl MT. Conformer Generation with OMEGA: Algorithm and Validation Using High Quality Structures from the Protein Databank and Cambridge Structural Database. J Chem Inf Model 2010;50(4):572–584.
4. OMEGA 3.1.2.2: OpenEye Scientific Software: Santa Fe, NM. http://www.eyesopen.com. 2020
5. Hawkins PCD, Skillman AG, Nicholls A. Comparison of Shape-Matching and Docking as Virtual Screening Tools. J Med Chem. 2007, 50 (1), 74–82.
6. ROCS 3.3.2.2: OpenEye Scientific Software, Santa Fe, NM. http://www.eyesopen.com
7. OpenEye Toolkits 2020.1.0 OpenEye Scientific Software, Santa Fe, NM. http://www.eyesopen.com.
8. Willett, P. Searching Techniques for Databases of Two- and Three-Dimensional Chemical Structures. J Med Chem 2005;48(13):4183–4199.
9. Jasial S, Hu Y, Vogt M, Bajorath J. Activity-Relevant Similarity Values for Fingerprints and Implications for Similarity Searching. F1000Res 2016; 5:Chem Inf Sci-591.
10. Berman HM, Westbrook J, Feng Z, Gilliland G, Bhat TN, Weissig H, Shindyalov I. N, Bourne PE. The Protein Data Bank. Nucleic Acids Res 2000;28(1):235–242.
11. Schrödinger Release 2020-1: Maestro; Schrödinger, LLC: New York, NY, 2020.
12. Porter NJ, Mahendran A, Breslow R, Christianson DW. Unusual Zinc-Binding Mode of HDAC6-Selective Hydroxamate Inhibitors. Proc Natl Acad Sci USA 2017;114(51):13459.
13. Sastry GM, Adzhigirey M, Day T, Annabhimoju R, Sherman W. Protein and Ligand Preparation: Parameters, Protocols, and Influence on Virtual Screening Enrichments. J Comput Aided Mol Des 2013;27(3):221–234.
14. Schrödinger Release 2020-1: Protein Preparation Wizard; Schrödinger, LLC: New York, NY, 2020.
15. Gantt SL, Joseph CG, Fierke CA. Activation and Inhibition of Histone Deacetylase 8 by Monovalent Cations. J Biol Chem 2010;285(9):6036–6043.
16. Gantt SM, Decroos C, Lee MS, Gullett LE, Bowman CM, Christianson DW, Fierke CA. General Base-General Acid Catalysis in Human Histone Deacetylase 8. Biochemistry 2016;55(5):820–832.
17. Bochevarov AD, Harder E, Hughes TF, Greenwood JR, Braden DA, Philipp DM, Rinaldo D, Halls, MD, Zhang J, Friesner RA. Jaguar: A High-Performance Quantum Chemistry Software Program with Strengths in Life and Materials Sciences. Int J Quantum Chem 2013;113(18):2110–2142.
18. Schrödinger Release 2020-1: Jaguar, Schrödinger, LLC, New York, NY, 2020.
19. Friesner RA, Banks JL, Murphy RB, Halgren TA, Klicic JJ, Mainz DT, Repasky MP, Knoll EH, Shelley M, Perry JK, et al. Glide: A New Approach for Rapid, Accurate Docking and Scoring. 1. Method and Assessment of Docking Accuracy. J Med Chem 2004;47(7):1739–1749.
20. Schrödinger Release 2020-1: Glide, Schrödinger, LLC, New York, NY, 2020.
21. Rush TS, Grant JA, Mosyak L, Nicholls A. A Shape-Based 3-D Scaffold Hopping Method and Its Application to a Bacterial Protein−Protein Interaction. J Med Chem 2005;48(5):1489–1495.
22. Kim JG, Kang D, Jang D. Preparation of α-Bromoacrylates: One-Pot Procedure for the Synthesis of Conjugated Acetylenic Carboxylates from Aldehydes with Ph_3P/Br_3CCO_2Et. Synlett 2008;3:443-447.
23. Augustine JK, Bombrun A, Venkatachaliah S, Jothi A. Titanium Mediated Olefination of Aldehydes with α-Haloacetates: An Exceptionally Stereoselective and General Approach to (Z)-α-Haloacrylates. Org Biomol Chem 2013;11(46):8065-8072.
24. Jiang B, Dou Y, Xu X, Xu M. A Novel and Convenient Protocol for Synthesis of α-Haloacrylates. Org Lett 2008;10(4):593-596.
